# Multifunctional Fluorescent Microgel‐Embedded Hydrogels for Temperature and X‐Ray Sensing

**DOI:** 10.1002/advs.202512518

**Published:** 2025-09-30

**Authors:** Hao Jiang, Luyao Wang, Li Jiang, Yajie Wang, Jinlong Zhang, Mingyuan Pan, Rui Hu, Jun Li, Chouwang Li, Chan Kyung Kim, Hong Chen, Liang Hu

**Affiliations:** ^1^ Key Laboratory of Polymeric Material Design and Synthesis for Biomedical Function College of Chemistry Chemical Engineering and Materials Science Soochow University Suzhou 215123 China; ^2^ State Key Laboratory of Radiation Medicine and Protection Collaborative Innovation Center of Radiological Medicine of Jiangsu Higher Education Institutions and School for Radiological and Interdisciplinary Sciences (RAD‐X) Soochow University Suzhou 215123 China; ^3^ Radiation Oncology Center Huashan Hospital Fudan University Shanghai 200040 China; ^4^ Department of Radiation Oncology The Affiliated Suzhou Hospital of Nanjing Medical University Suzhou Municipal Hospital Suzhou 215000 China; ^5^ School of Chemistry and Chemical Engineering North University of China Taiyuan 030051 China; ^6^ Department of Chemistry and Chemical Engineering Inha University Incheon 22212 South Korea

**Keywords:** aggregation‐induced emission luminogen, ratiometric fluorescent, sensing, stimuli‐responsive hydrogel, support vector machine regression

## Abstract

Stimuli‐responsive aggregation‐induced emission luminogen (AIEgen)‐based hydrogels exhibit tunable fluorescent signals in response to external stimuli, providing a suitable platform for sensing. As the largest sensory organ in the body, the response of skin temperature and X‐ray dosage is crucial in predicting and diagnosing a range of diseases, and effective cancer radiotherapy. In this study, dual‐responsive hydrogels are prepared by incorporating ratiometric fluorescent AIEgen‐based microgels into polyvinyl alcohol (PVA). Experimental and molecular dynamics (MD) simulation results indicate that the AIEgen‐based microgels enhance the mechanical and adhesive strength of the PVA hydrogels while maintaining good biocompatibility. Variations in temperature or X‐ray exposure affect the chemical structure of the co‐monomer or disulfide/diselenium‐containing crosslinkers, which influences the molecular motion of the synthesized AIEgen; the other fluorescent molecules are unaffected. Consequently, the ratiometric fluorescent signals emitted by the AIE microgel‐embedded hydrogels exhibit spectral and visual variations in response to changes in temperature and X‐ray exposure. Applying support vector machine (SVM) regression, the hydrogel can achieve improved spectral accuracy. The hydrogel is shown to accurately sense skin temperature and effectively map radiotherapy dose levels.

## Graphical abstract

In this study, the application of a multifunctional fluorescent hydrogel based on AIE microgels, integrated with machine‐learning algorithms for the detection of temperature and X‐rays is prestented. This innovative platform not only simultaneously elevates temperature and X‐ray sensing performance but also preserves outstanding mechanical robustness and excellent biocompatibility.

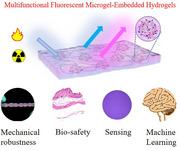

## 1 Introduction

Hydrogels cross‐linked with hydrophilic polymers exhibit a distinctive network structure filled with water.^[^ ^1^, ^2^, ^3^, ^4^, ^5^, ^6^ ^]^ These hydrogels often incorporate fluorophores, which enhance associated optical properties.^[^ ^7^, ^8^, ^9^, ^10^ ^]^ Conventional fluorophore components tend to aggregate, which can result in fluorescence quenching due to hydrogel confinement effects. Aggregation‐induced emission luminogens (AIEgens) were first reported a decade ago, offering a solution to the aggregation‐induced quenching of conventional chromophores.^[^ ^11^ ^]^ Moreover, AIEgens exhibit superior photostability, photosensitizing capability, and enhanced brightness. As a result, AIEgens have emerged as viable alternatives for the development of fluorescent hydrogels.^[^ ^12^, ^13^ ^]^ In particular, stimuli‐responsive AIEgen‐based hydrogels generate tunable fluorescent signals in response to external stimuli, such as temperature, pH, ions, and light.^[^ ^14^, ^15^ ^]^ Therefore, stimuli‐responsive AIEgen‐based hydrogels are the focus of appreciable research due to the wide‐ranging potential applications in sensing, actuators, and information encryption.^[^ ^16^, ^17^, ^18^ ^]^

The skin is the largest sensory organ in the body. Skin temperature is a critical indicator used in the prediction and diagnosis of various diseases, including cardiovascular diseases, severe fever, and infection.^[^ ^19^, ^20^ ^]^ Conventional thermometers are inherently limited in sensitivity and stability, which has driven widespread interest in fluorescence‐based alternatives.^[^ ^21^ ^]^ Poly(N‐isopropyl acrylamide) (pNIPAm) is widely used as a temperature‐responsive polymer that transitions from a hydrophilic (random coil conformation) to a relatively hydrophobic (globular conformation) state as the temperature is increased above the lower critical solution temperature (LCST, ≈32 C). Temperature‐stimuli responsive AIEgen‐based hydrogels have been extensively studied.^[^ ^22^, ^23^, ^24^, ^25^ ^]^ Unfortunately, most reported examples centered on tetraphenylethylene derivatives, and displayed divergent response behaviors and low sensitivity, making them inadequate for skin‐temperature monitoring (Table S1, Supporting Information).

In addition, an evaluation and determination of skin ionizing radiation (e.g., X‐rays) doses is of utmost importance, especially for cancer patients undergoing radiotherapy. This is because skin is typically the first tissue that is damaged under ionizing radiation.^[^ ^26^, ^27^, ^28^, ^29^ ^]^ Typically, a dose of several tens of Gy in 2–10 Gy (J Kg^−1^) fractions of radiation is used for cancer treatment.^[^ ^30^, ^31^, ^32^ ^]^ Monitoring skin radiation doses can enable a prediction of radiation‐induced skin injuries and inform suitable skin protection strategies. Moreover, monitoring skin radiation doses allows for adjustments in radiotherapy parameters when needed. However, it is still a major challenge.^[^ ^33^ ^]^ Conventional dosimeters exhibit an energy/dose rate dependence and require complicated preparation/operation procedures with extended detection periods. Moreover, these dosimeters are solid and unsuitable for skin dosing. Our group has developed microgels for X‐ray dosimetry, however, the majority of these systems can only effectively measure 1D X‐ray doses. ^[^ ^34^, ^35^, ^36^ ^]^ EBT‐3 films can measure absorbed doses in the skin, but the readout signals must be obtained 24 h after radiation exposure. In addition, the virtual treatment planning system (TPS) often provides an inaccurate skin dose estimation.^[^ ^37^ ^]^ Currently, there is no established flexible hydrogel‐based sensor for skin radiation dosimetry, although AIEgen‐based materials have been studied for use as scintillators.^[^ ^38^ ^]^

**Scheme 1:**
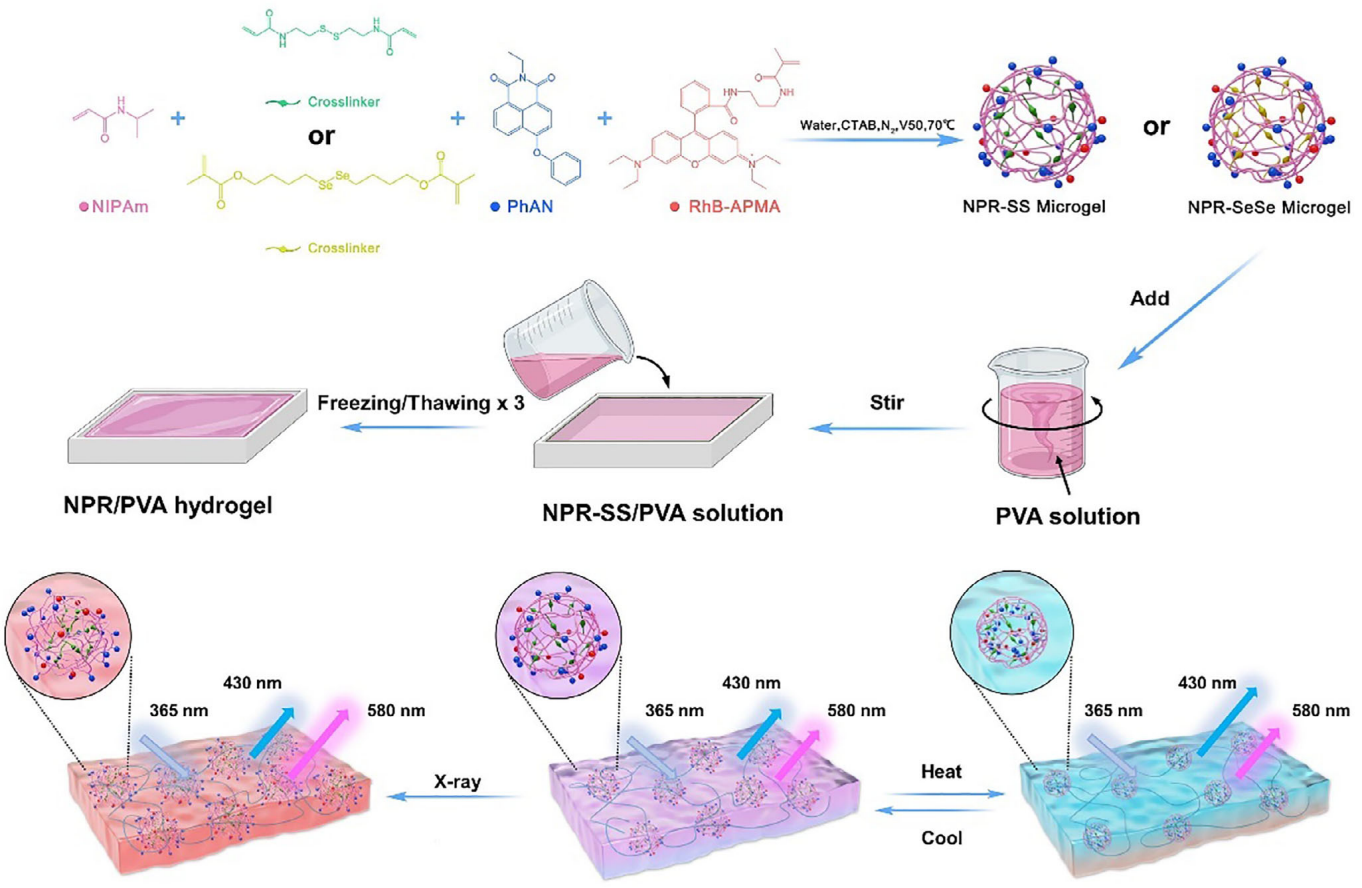
A schematic illustration of the preparation of dual responsive AIE microgel‐embedded hydrogels for temperature and X‐ray sensing.

Herein, we report the first use of 4‐phenoxy‐N‐allyl‐1,8‐naphthalenedicarboximide (PhAN)‐based multifunctional hydrogel with machine‐learning algorithms for temperature and X‐ray decoding, with fast detection, high sensitivity, and accuracy. AIEgen‐based microgels were first synthesized by free radical polymerization in the presence of NIPAm (as primary monomer), PhAN (as an AIE co‐monomer, λ_ex_/λ_em_: 365/430 nm), RhB derivative (as an inert fluorescent co‐monomer, λ_ex_/λ_em_: 365/580 nm), and X‐ray‐responsive crosslinkers (i.e., N, N'‐bis(acryloyl)cystamine, BAC; diselenediylbis (butane‐4,1‐diyl) bis(2‐methylacrylate, BMASe). Microgels provide a confined network for AIEgens and can respond to external stimuli markedly faster than their bulk‐hydrogel counterparts. Next, the AIEgen‐based microgels were introduced into polyvinyl alcohol (PVA), generating a ratiometric fluorescent hydrogel. Without PVA, the microgel suspension would not be mechanically robust, rendering it unsuitable for reliable body‐temperature and X‐ray sensing. When compared with the pure PVA hydrogel, the AIEgen‐based microgels contributed to improved mechanical properties. Applying an external temperature or X‐ray stimulus, the microgel chemical conformation was altered, influencing intramolecular motion and generating ratiometric fluorescent signals associated with the AIEgen that can be detected visually and spectrally (**Scheme** **1**). We have explored the application of machine learning models to overcome potential spectral inaccuracy, including linear and quadratic regressions, as well as random forest, K‐nearest neighbors (KNN), and support vector machine (SVM) regressions. Our platform simultaneously elevates temperature and X‐ray sensing performance while preserving outstanding mechanical robustness and excellent biocompatibility (Tables S1 and S2, Supporting Information).

## 2 Results and Discussion

### 2.1 Synthesis of PhAN

**Figure 1:**
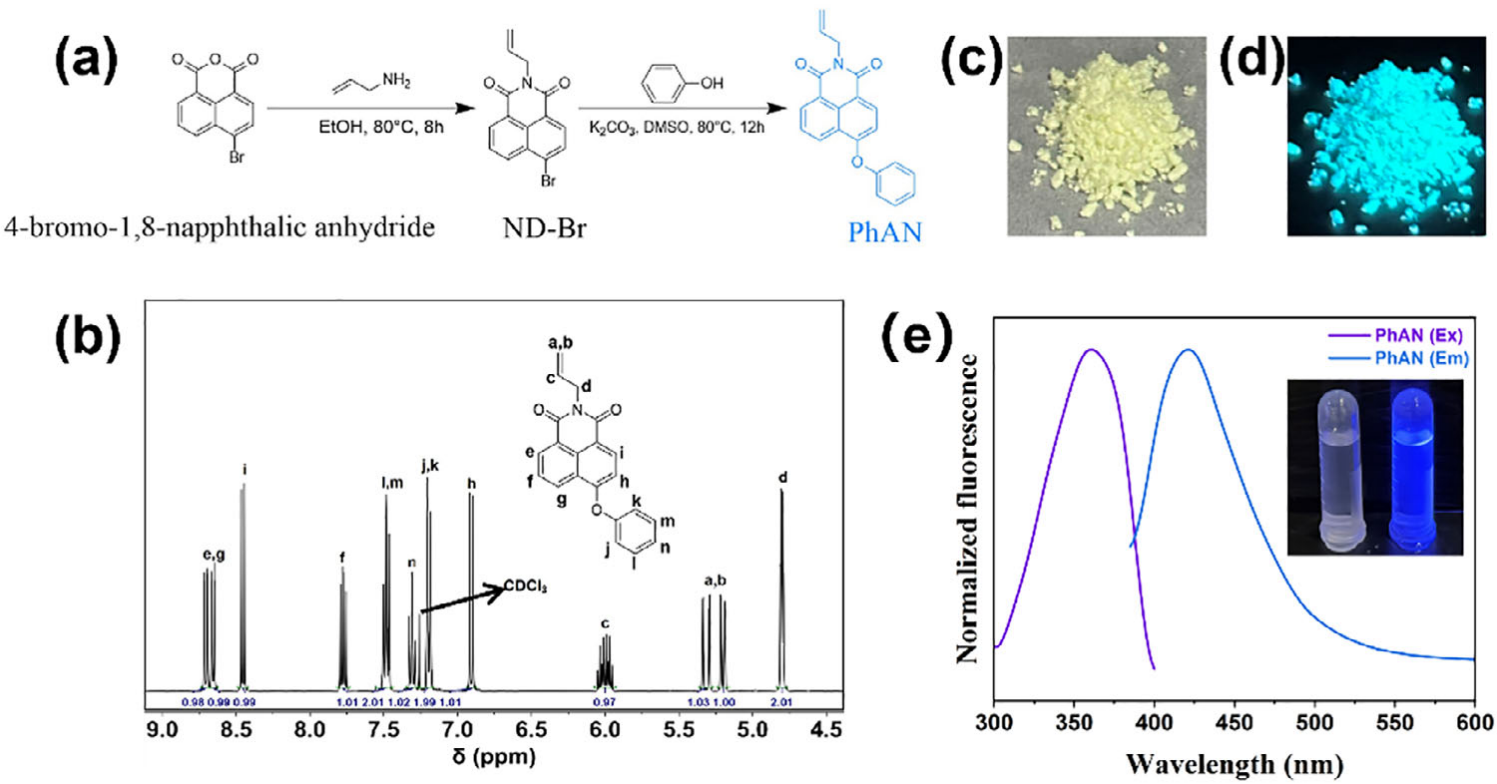
Properties of PhAN: a) synthesis routine, b) ^1^H NMR spectrum, in CDCl_3_, digital photographs under c) white and d) UV light, e) fluorescence spectra (insert THF solvent on the left and PhAN in THF on the right).

4‐Bromo‐1,8‐naphthalic anhydride was reacted with allylamine hydrochloride with heating in an anhydride amidation reaction, yielding 4‐bromo‐N‐allyl‐1,8‐naphthalenedicarboximide (ND‐Br) as evidenced by ^1^H nuclear magnetic resonance (^1^H NMR) spectrum in Figure S1 (Supporting Information). Subsequently, PhAN was synthesized via a base‐catalyzed nucleophilic substitution reaction between ND‐Br and phenol with heating (**Figure** **1**a). The chemical structure of PhAN was confirmed by ^1^H NMR and electron spray ionization‐mass spectrometry (ESI‐MS) analysis. As shown in Figure 1b, the ^1^H NMR spectrum exhibits signals due to protons associated with C*H* = C*H*_2_ at 5.20 (5.32) and 6.00 ppm. Protons associated with the benzene and naphthalene rings are observed at 6.91–8.71 ppm. The ratio of the integral area of each proton peak in the ^1^H NMR spectrum aligns with the theoretical ratio of hydrogen atoms in PhAN. The ESI‐MS spectrum (Figure S2, Supporting Information) exhibits a [M+H]^+^ value of 330.3381 that closely matches the theoretical value (330.1130). The PhAN powders appear yellow under white light (Figure 1c). When exposed to 365 nm UV irradiation, the powders emit a bright blue fluorescence (Figure 1d). In addition, when the PhAN monomer was dissolved in tetrahydrofuran (THF), the resultant PhAN solution retained its fluorescent properties, with optimal excitation and emission wavelengths of 362 and 423 nm, respectively (Figure 1e).

### 2.2 Synthesis of Poly(NIPAm‐co‐PhAN‐co‐RhB‐APMA) Microgels

**Figure 2:**
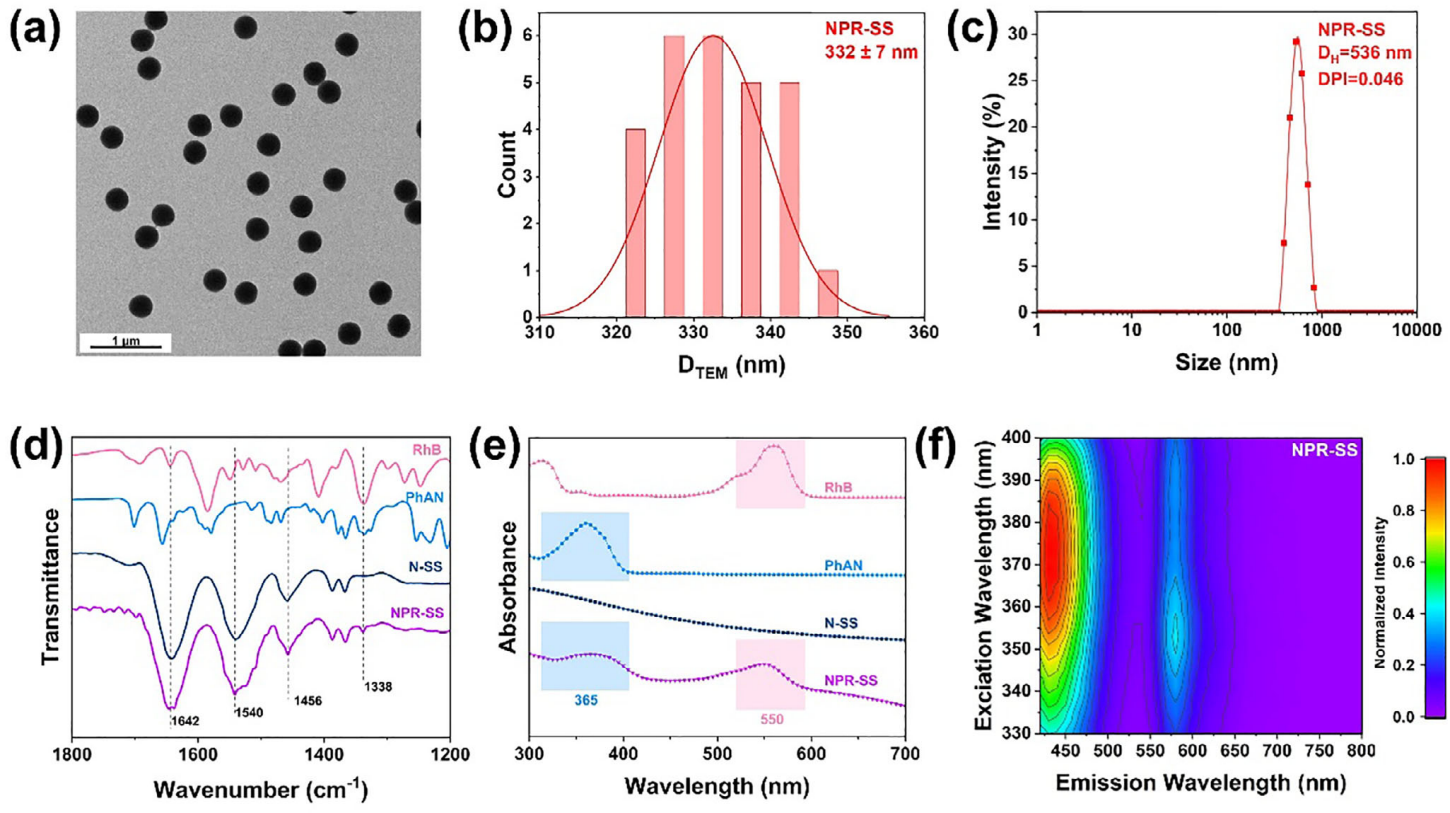
Properties of the NPR‐SS microgel: a) TEM image, b) statistics analysis of D_TEM_, c) DLS, d) FTIR, e) UV–vis, and f) 2D fluorescence emission/excitation spectra.

Poly(NIPAm‐co‐PhAN‐co‐RhB‐APMA) (denoted as NPR‐SS) microgel was synthesized via precipitation polymerization using NIPAm, PhAN, and RhB‐APMA (APMA: N‐(3‐aminopropyl)methacrylic acid hydrochloride) as co‐monomers, with BAC as the cross‐linking agent (Scheme 1). The transmission electron microscopy (TEM) image and analysis have revealed that the NPR‐SS microgels exhibit a regular spherical structure, with a diameter (D_TEM_) of 332 ±7 nm (**Figure** **2**a,b). Dynamic light scattering (DLS) measurement shows that the addition of deionized (DI) water resulted in a swelling of the NPR‐SS, with a hydrodynamic diameter (D_H_) of 536 nm and a narrow D_H_ distribution (polydispersity index, PDI = 0.046), demonstrating homogeneity (Figure 2c). The Fourier transform infrared spectroscopy (FTIR) spectra of the NPR‐SS microgels exhibit characteristic peaks at 1642 and 1540 cm^−1^ due to amide N─H and C═O stretching vibrations, respectively (Figure 2d).^[^ ^39^ ^]^ The characteristic peak at 1456 cm^−1^ is attributed to a ─CH_3_ bending vibration associated with the isopropyl group of NIPAm. As a control, polyNIPAm microgel was prepared using BAC as the crosslinker (denoted as N‐SS microgel). When compared with the FTIR spectrum of N‐SS microgel, the NPR‐SS microgel exhibited a characteristic peak at 1338 cm^−1^, which may be ascribed to the C‐C stretching vibration of the aromatic backbone in PhAN and RhB‐APMA. Compared to the N‐SS microgel, the NPR‐SS microgel exhibits distinct UV–vis absorption peaks at 365 and 550 nm, respectively, which are attributed to the aromatic conjugated structure of RhB and PhAN (Figure 2e). The excitation and emission fluorescence spectra of the NPR‐SS microgel are shown in Figure 2f. The excitation wavelengths of the NPR microgels are in the range of 360–380 nm. The maximum emission wavelengths at ≈430 and ≈580 nm correspond to the PhAN and RhB‐APMA moieties, respectively.

**Figure 3:**
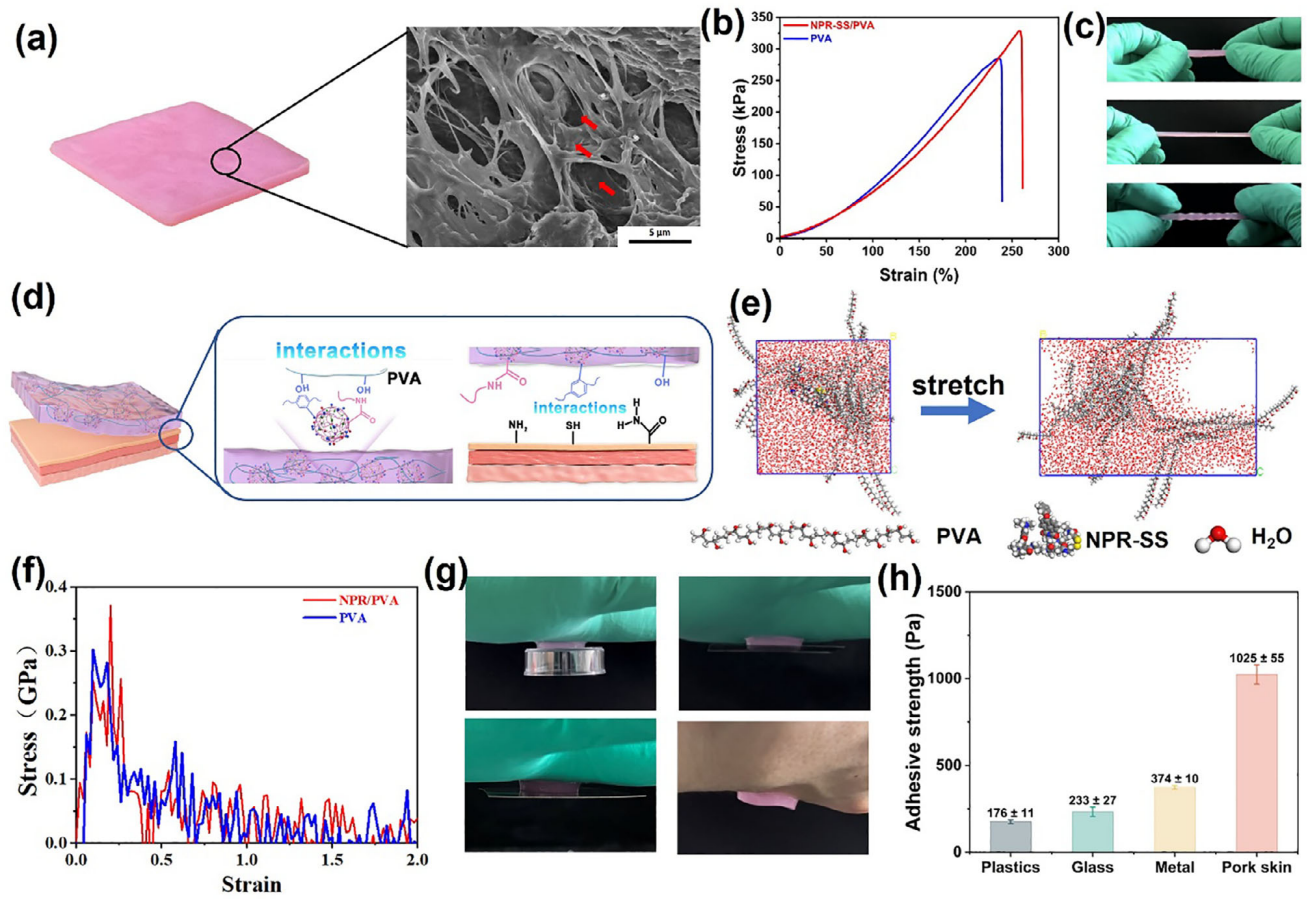
Properties of the NPR‐SS/PVA hydrogel: a) digital and SEM image, red arrows indicate NPR‐SS microgels, b) σ‐ε curve, c) digital photos of the NPR‐SS/PVA hydrogel under stretching and twisting. d) A schematic illustration of molecular interactions among PVA, the NPR‐SS microgel, and substrates. e) Snapshots showing the MD simulations before and after being stretched to a strain of 200%. f) Simulated σ‐ε curve, g) digital images showing adhesion, h) adhesive strength to different substrates. Each data point is the average of 3 measurements, with the error bars showing the standard deviation.

The NPR‐SS/PVA hydrogel was produced using a freeze‐thaw method, where the resultant NPR‐SS/PVA hydrogel appeared pink (**Figure** **3**a). The 3D porous structure of NPR‐SS microgels embedded in the PVA matrix is apparent with evidence of surface wrinkles. Suitable hydrogel mechanical properties and cytotoxicity are essential for effective application. As shown in Figure 3b, the fracture stress (σ_f_) and strain (ε_f_) of the NPR‐SS/PVA hydrogel are 318.1±13.1 kPa and 257.7%±11.5%, respectively (*n* = 3 independent samples). The Young's modulus and rupture energy reach 123.8±3.0 kPa, and 335.8±32.3 kJ^−1^ m^−3^, respectively. Consequently, the NPR‐SS/PVA hydrogel can be stretched and twisted (Figure 3c). The mechanical properties of the NPR‐SS/PVA hydrogel are superior to the pure PVA hydrogel, which shows 284.2±22.3 kPa of σ_f_ and 239.7%±9.6% of ε_f_, respectively. Furthermore, the NPR‐SS/PVA hydrogel can also withstand compression (369.7±4.8 kPa) without breaking, which is superior to the pure PVA hydrogel (354.7±10.1 kPa) as well (Figure S3, Supporting Information). This can be attributed to the introduction of the NPR‐SS microgels, which facilitated hydrogen bonding and π‐OH intra‐/interactions between NPR‐SS and PVA (Figure 3d). The molecular dynamics (MD) simulation using forcite modules in the Materials Studio software was undertaken to evaluate the uniaxial tensile testing of the NPR‐SS/PVA and PVA hydrogels. The simulation visual images can be seen in Figure 3e and Figure S4 (Supporting Information). Increasing the strain led to the generation of voids, and finally, fractures occurred. As presented in Figure 3f, σ reaches a maximum at the elastic stage, where a scission of the physical cross‐linking bonds results in a sudden drop in stress. Subsequently, the material undergoes plastic deformation. The MD simulations have demonstrated an enhanced σ for the NPR‐SS/PVA hydrogel relative to the PVA hydrogel, although the simulated values of σ differ from the experimental data. As the MD simulations are at the microscopic level, the resultant σ exceeds the macroscopic experiments.

The NPR‐SS/PVA hydrogel can adhere to a variety of substrates, including plastic sheets, glass, titanium sheets, and pig skin (Figure 3g). The shear adhesion test established the following adhesion strengths: 176±11 Pa (plastic), 233±27 Pa (glass), 374±10 Pa (titanium sheet), and 1025±55 Pa (pig skin, Figure 3h). This adhesion force is due to interactions between the substrates and the NPR‐SS/PVA hydrogel, including hydrogen bonding, π‐thiol, π‐amine bonds, and so on.

**Figure 4:**
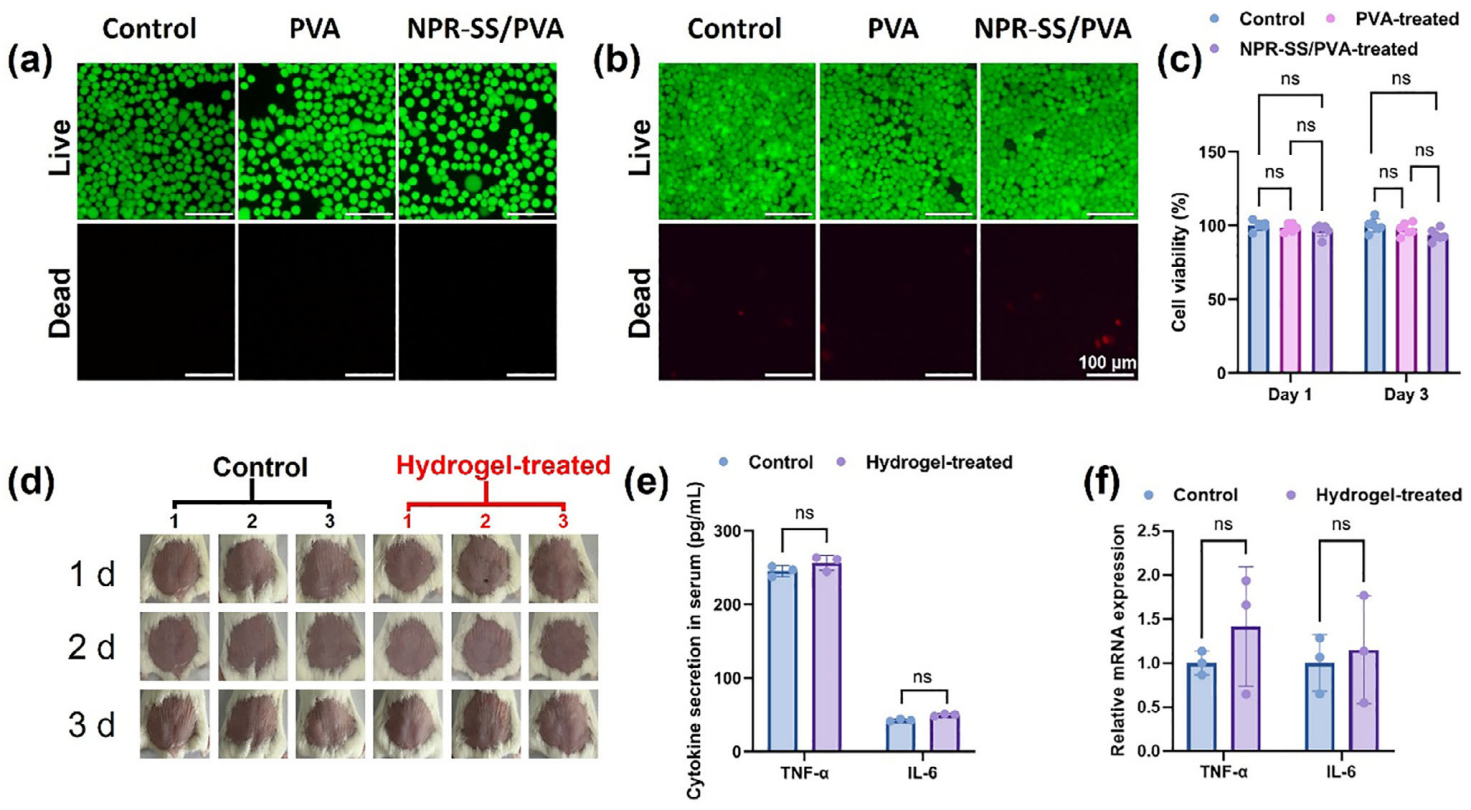
The biocompatibility of the NPR‐SS/PVA hydrogel: a) Live/dead images of HeLa cells incubated with the specimens for a) 1, b) 3 days, Scale bars = 100 µm. c) Cell viability of the NPR‐SS/PVA hydrogel. Each data point is the average of 5 measurements, with the error bars showing the standard deviation. “ns” denotes no significance. d) Digital images of skin treated by the NPR‐SS/PVA hydrogel. TNF‐α and IL‐6 levels in e) serum and f) tissue. Each data point in panels *e and f* is the average of 3 measurements, with the error bars showing the standard deviation. “ns” denotes no significance.

The biocompatibility of the hydrogel is crucial, as it must adhere to facial skin during sensing. Therefore, a comprehensive series was carried out, including cytotoxicity assessments, skin irritation evaluations, histopathological analysis of the skin, hematological examinations, and biochemical tests. The cytotoxicity of NPR‐SS/PVA hydrogels was assessed using HeLa cells. As illustrated in **Figure** **4**a, live/dead staining images clearly show that the number and morphology of HeLa cells incubated with NPR‐SS/PVA hydrogels are comparable to those in the control and PVA hydrogel groups after 1 and 3 days. After treatment for 1 day, the cell viability of HeLa cells was 97.51%, compared with 98.42% for the PVA hydrogel. Following 3 days of co‐culture, the NPR‐SS/PVA hydrogels maintained a high level of cell viability (>93%). In vivo biosafety was further evaluated in female BALB/c mice. First, skin irritation potential was assessed according to the standards for the biological evaluation of medical devices. Compared with saline control dressing, the NPR‐SS/PVA hydrogel does not induce erythema or edema after dressing removal (Figure 4d; Figure S5, Supporting Information). Hematoxylin and eosin (H&E) and Masson‐stained sections reveal that the NPR‐SS/PVA hydrogel does not cause visible damage to skin tissues (Figure S5, Supporting Information). The epidermis at the application site remain intact, exhibiting no leukocyte infiltration, vascular congestion, or edema after three days, showing a negligible inflammatory response. Masson's staining further confirms that the NPR‐SS/PVA hydrogel has no impact on collagen production. Additionally, the NPR‐SS/PVA hydrogel does not increase TNF‐α and IL‐6 levels in serum or tissue, as shown in Figure 4e,f. Together, these findings demonstrate that the NPR‐SS/PVA hydrogel exhibits excellent biocompatibility at the cytological, histological, and hematological levels, showcasing its translational potential for clinical applications.

**Figure 5:**
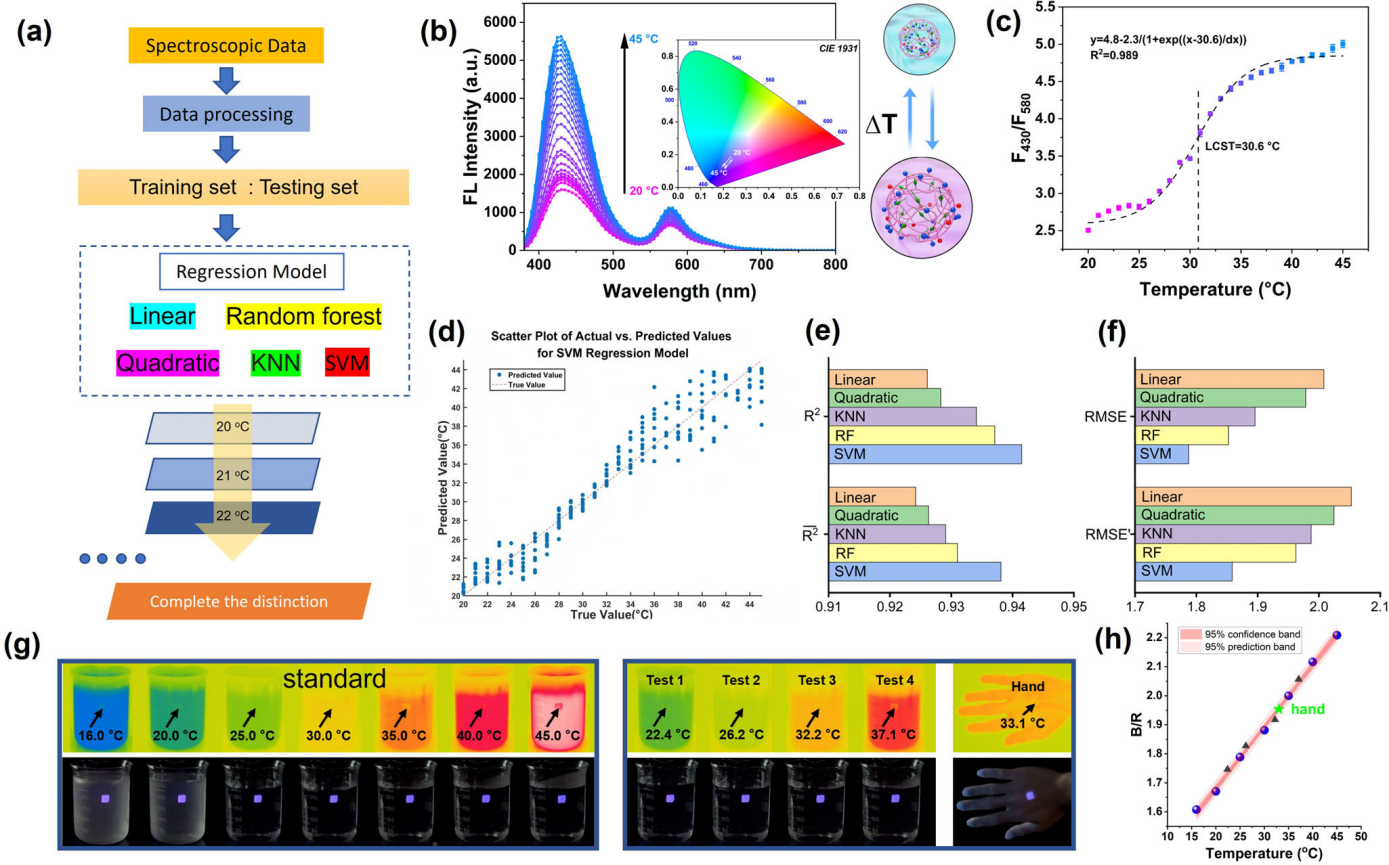
Temperature responsivity of the NPR‐SS/PVA hydrogel: a) schematic illustration of the regression process, change in b) FL spectra and CIE image of the hydrogel and schematic illustration of the embedded NPR‐SS microgels responding to temperature, c) F_430_/F_580,_ and d) SVM regression of the NPR‐SS/PVA hydrogel as a function of increasing temperatures. Each data point in panel *c* is the average of 60 measurements, with the error bars showing the standard deviation. e) R^2^, $\bar{R^{2}}$ and f) RMSE, RMSE’ of different regression models. g) Digital and fluorescent images of the NPR‐SS/PVA hydrogel attached to a beaker and a volunteer's hand. h) B/R values from panel *g* at different temperatures. ●: standard samples, ▲: tests from the beaker, ★: test from the hand.

We next moved to study the NPR‐SS/PVA hydrogel response to temperature, applying different regression models to experimentally acquired signals (**Figure** **5**a). As shown in Figure 5b, as the temperature is increased from 20 to 45 °C in increments of 1 °C, the fluorescence spectra change immediately. The fluorescence intensity at 430 nm (F_430_) of NPR‐SS/PVA gradually increases relative to that at 580 nm (F_580_). The CIE 1931 plot also shows that the fluorescence emission peaks of the NPR‐SS/PVA hydrogel gradually blueshift as the temperature increases. Quantitatively, it can be seen that the fluorescence intensity ratio (F_430_/F_580_) exhibits an exponential increase from 2.5 to 5.1, which can be fitted to the expression:

(1) $$F_{430}/\;F_{580}=4.8-2.3/\left(1+\exp\left(\left(x_{\mathrm{temp}}-30.6\right)/dx_{\mathrm{temp}}\right)\right)$$

where *x_temp_* represents temperature (Figure 5c).

Ratiometric fluorescence detection is renowned for its rapid response, exceptional sensitivity, and accuracy. In contrast to prior literature, our hydrogel demonstrates markedly superior sensitivity, attaining a remarkable 0.1 °C^−1^ (Table S1, Supporting Information).

To understand the underlying mechanism of the temperature‐responsive fluorescence, the temperature‐dependent FTIR spectra of the NPR‐SS microgel solution (in D_2_O) were measured. In the aqueous solution, the amide I band shifts from 1642 cm^−1^ (solid‐state FTIR in Figure 2d) to 1621 cm^−1^, indicating that polymer chains form the H bonds and /or are hydrated (Figure S6, Supporting Information). Comparing spectra at 25 and 50 °C, noticeable shifts in the peak positions of several vibrational modes can be observed. Upon heating to 50 °C, the intensity of the amide I band is enhanced. Meanwhile, peaks assigned to the isopropyl groups are lower at 50 °C than those at 25 °C. These changes reflect the coil‐to‐globule transition of pNIPAm chains, which is consistent with earlier reports.^[^ ^40^ ^]^ Importantly, an increase in temperature reduces NPR‐SS microgel volume, with an accompanying decrease in D_H_ from ≈515 (20 °C) to ≈375 nm (45 °C, Figure S7, Supporting Information). Dehydration of the microgel matrix suppresses the non‐radiative decay pathway of PhAN, whereas the elevated temperature simultaneously activates the rotational and vibrational modes of both PhAN and RhB (Figure S8, Supporting Information). These effects synergistically boost PhAN fluorescence while marginally attenuating that of RhB, as evident in Figure 5b. Fitting the fluorescence at different temperatures using the Boltzmann equation, the calculated lower critical solution temperature (LCST_FL_) of NPR‐SS/PVA hydrogels is 30.6 °C, which agrees with the LCST_FL_ as well as LCST_DLS_ of NPR‐SS microgels in solution (Figure S9, Supporting Information). It should be noted that the NPR‐SS/PVA hydrogel shows reversible temperature responsivity and storage stability. As the temperature decreases, F_430_/F_580_ returns to its initial value. Following storage for three days, NPR‐SS/PVA hydrogel still could respond to temperature, although the fluorescence intensity was slightly increased, due to hydrogel dehydration (Figure S10, Supporting Information).

Conventional linear regression is often unable to adequately capture complex, nonlinear relationships between acquired signals and the analytes of interest. Machine learning‐based nonlinear regression models can automatically learn the mapping between input features and target variables without the need for manual derivation of mathematical expressions, offering superior performance with intricate datasets. In this study, we explored several nonlinear regression machine learning models (e.g., KNN, random forest (RF), and SVM). In order to enhance model performance and generalization, we normalized the data and randomly split the dataset into a training set (70%) and a test set (30%). We evaluated the prediction accuracy and fitting performance of each model using the root mean square error (RMSE) and coefficient of determination (R^2^). As shown in Figure S11 (Supporting Information), linear regression and quadratic regression were limited in addressing complex nonlinear relationships. However, KNN, RF, and SVM delivered significantly better performance (Figure S11, Supporting Information; Figure 5d). Specifically, the SVM model achieved superior prediction accuracy with the highest R^2^ (0.9415) and lowest RMSE (1.7875), possibly due to an insensitivity to local data structures and noise (Figure 5e, f). Furthermore, in 10‐fold cross‐validation experiments, SVM consistently demonstrated the best generalization capability, recording the highest $\bar{R^{2}}$ (0.9381) as well as the lowest RMSE' (1.8586). Furthermore, the residual scatter plots and histograms reveal that the SVM model delivers the best overall performance (Figure S12, Supporting Information). When applied to the test set, the SVM model produces more residuals close to zero with a more uniform distribution, with markedly fewer error samples compared to the other models. Meanwhile, the residual histogram of the SVM model features a typical normal distribution, indicating that the model can capture the underlying data pattern accurately, with negligible systematic bias, minimal extreme errors, and the strongest stability among all tested models. Compared to the SVM model, the residual histograms of linear and quadratic regression models exhibit significant deviations from the normal distribution, indicating that both models fail to capture the nonlinear relationship between temperature and spectral data. In terms of residual characteristics, both models lack central tendency and exhibit evident systematic bias, rendering them unsuitable for this dataset. In addition, RF and KNN models show comparable performance but are less accurate than the SVM model, with a higher propensity for moderate to large errors and extreme deviations.

The NPR‐SS/PVA hydrogel can also sense temperature by fluorescent image analysis. In the initial test, the NPR‐SS/PVA hydrogel was attached to a beaker filled with water. As the temperature was increased from 16 to 45 °C, the fluorescence images were recorded, and the associated RGB values were analyzed using Photoshop software (Figure 5g). It was found that the B/R value increased as a function of increasing temperature, expressed by (Figure 5h):

(2) $$B/R=0.02\times x_{temp}+1.3\;\left(\;R^{2}=0.998\right)$$

The hydrogel was subsequently attached to a beaker filled with water at unknown temperatures, and the fluorescence images were captured. The B/R values were then substituted into Equation 2 to determine the temperature. It was found that the temperatures measured using the hydrogels were consistent with those recorded by thermal imaging (Figure 5h).

In addition, the hydrogel was capable of measuring skin temperature when applied to a volunteer's hand. Applying the same method, the skin temperature was measured at 32.9 °C, close to that recorded (33.1 °C) in thermal imaging measurement. These results establish viable use of the NPR‐SS/PVA hydrogel in sensing skin temperature.

### 2.3 X‐Ray Dose Sensing

**Figure 6:**
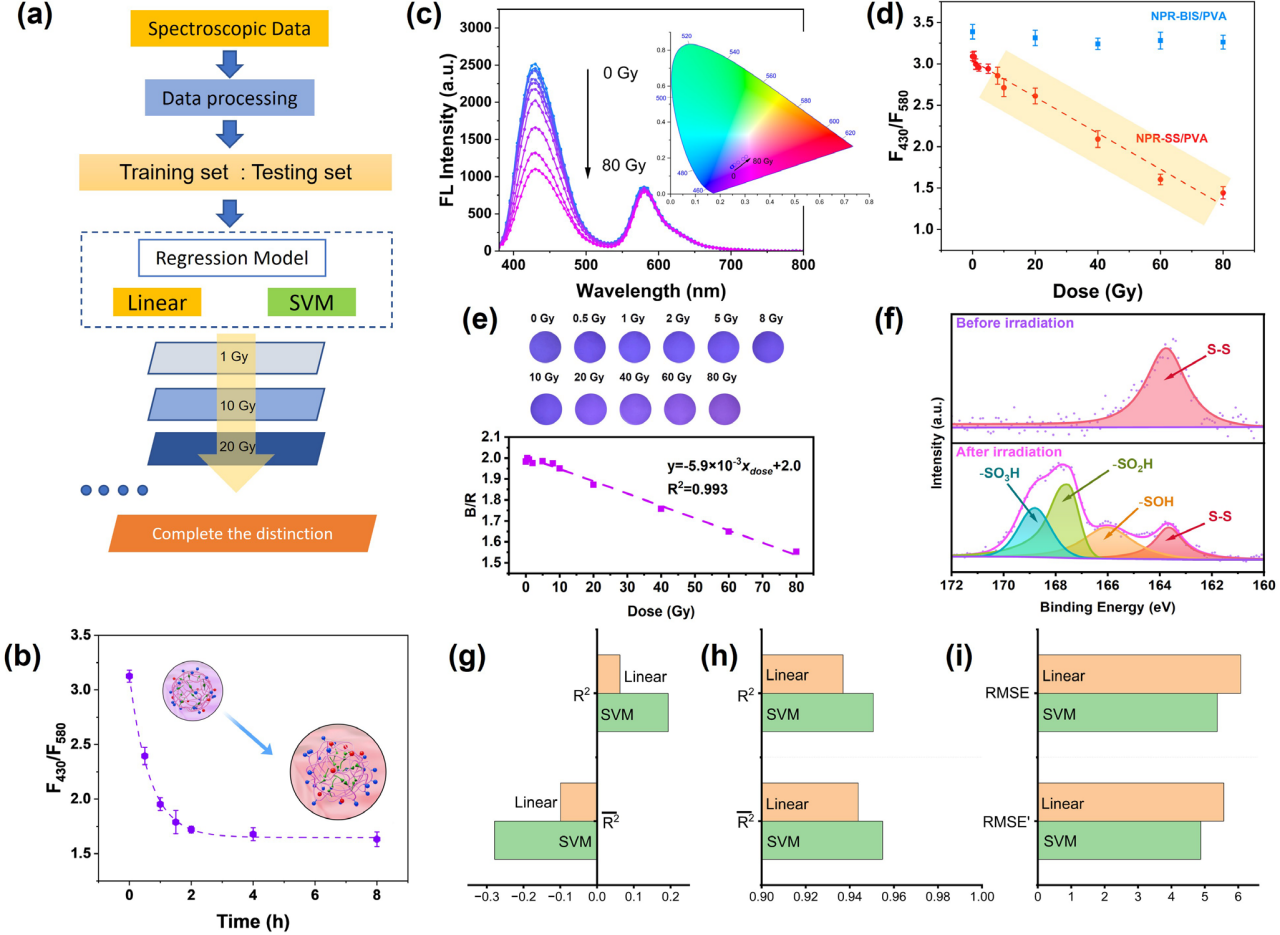
Properties of the NPR‐SS/PVA hydrogel responding to X‐rays: a) schematic illustration of regression process. b) kinetics of F_430_/F_580_ after X‐ray irradiation and schematic illustration of the embedded NPR‐SS microgels responding to X‐rays. Each data point is the average of 3 measurements, with the error bars showing the standard deviation. Change in c) FL intensity and CIE image, d) F_430_/F_580_, e) fluorescence images and B/R values of hydrogels as a function of increasing X‐ray doses. Each data point in panel *d* is the average of 60 measurements, with the error bars showing the standard deviation. Each data point in panel *e* is the average of three measurements, with the error bars showing the standard deviation. f) XPS spectra of NPR‐SS microgels before and after irradiation. R^2^ and $\bar{R^{2}}$ analysis of the NPR‐SS/PVA hydrogel under g) 0 – 8 Gy and h) 10–80 Gy. i) RMSE and RMSE’ analysis of the NPR‐SS/PVA hydrogel under 10–80 Gy.

The capability of NPR‐SS/PVA hydrogels to sense X‐ray doses has been assessed (**Figure** **6**a). Kinetics tests were conducted to quantify the response of NPR‐SS microgels to X‐rays. As shown in Figure 6b, when exposed to 60 Gy X‐rays (225 kVp, 26.5 Gy min^−1^), the F_430_ of NPR‐SS microgels exhibits a sharp decrease, whereas F_580_ is slightly altered with a resultant decrease in F_430_/F_580_. Following 2 h post‐irradiation, the change in F_430_/F_580_ is stable for at least 8 h. Next, the NPR‐SS/PVA hydrogel was prepared and utilized to sense X‐ray doses by monitoring F_430_ and F_580_ after 2 h post‐irradiation. The hydrogel was irradiated by X‐rays in the vertical direction. As shown in Figure 6c, an increase in X‐ray radiation dose from 0 to 80 Gy is accompanied by a gradual decrease in F_430_ and a slight change in F_580_. Accordingly, the CIE 1931 plot shows the color red‐shifts. As a control, the fluorescence signals of the NPR‐BIS/PVA hydrogel are invariant under 0–80 Gy X‐ray irradiation, indicating that X‐rays have no effect on PhAn and RhB (Figure 6d; Figure S13, Supporting Information). In the case of the NPR‐SS/PVA hydrogel, F_430_ /F_580_ decreases linearly with a sensitivity of 0.02 Gy^−1^:

(3) $$F_{430}/F_{580}=-0.02\;\times \;x_{dose}+2.9\;\left(\;R^{2}=0.989\right)$$

where *x*_dose_ represents the X‐ray irradiation dose. The calculated lower limit of detection (LOD) of the NPR‐SS/PVA hydrogel is 4.9 Gy.

The fluorescent images of the NPR‐SS/PVA hydrogel switches from blue to purple as a function of increasing X‐ray dose (Figure 6e). The B/R value decreases as a function of increasing X‐ray doses, where:

(4) $$B/R=-5.9\times 10^{-3}\;\times x_{\mathrm{dose}}+2.0\;\left(\;R^{2}=0.993\right)$$

In order to gain an understanding of the underlying mechanism of X‐ray dose sensing, X‐ray photoelectron spectroscopy (XPS) and Raman analysis of NPR‐SS microgels were conducted before and after irradiation. The XPS analysis reveals an S2p_3/2_ peak due to the disulfide bond at 163.5 eV before irradiation. Exposure to 60 Gy X‐rays results in radiolysis of the water in the hydrogel, generating •OH radicals that reacted with the disulfide bonds via single‐electron oxidation, producing–S• and –S^+^. These products can further react with O_2_ and reactive oxygen species (ROS), forming a series of sulfur oxyacids, including sulfenic acid (–SOH), sulfinic acid (–SO_2_H), and sulfonic acid (–SO_3_H). Accordingly, signals due to ‐SOH (166.1 eV), ‐SO_2_H (167.9 eV), and ‐SO_3_H (169.0 eV) are observed in the XPS spectrum (Figure 6f). Meanwhile, the characteristic peak due to the disulfide bond is present in the Raman spectrum at 505 cm^−1^, decreasing in intensity after irradiation (Figure S14, Supporting Information). Based on Flory's theory of swelling, scission of the disulfide bond decreases the mole fraction of the BAC crosslinker, triggering a network swelling of microgels. Consequently, the D_H_ of the NPR‐SS microgels increases from ≈540 to ≈710 nm (Figure S15, Supporting Information). An increased volume of NPR‐SS microgels promotes the active PhAN intramolecular motion, which accelerates the non‐radiative decay of excitons with a consequent decrease in F_430_. In order to facilitate clinical application, we investigated the influence of dose rate on sensing. The X‐ray dose rate was modulated by adjusting the tube current. As shown in Figure S16 (Supporting Information), when varying the dose rate from 1.941 to 14.21 Gy min^−1^, the maximum difference of dose rate‐independent factor (DRIF) is 105±3.29%, meeting clinical requirements. This dose rate independence surpasses that of both Geiger‐Müller tubes and ionization chamber dosimeters.

We have compared the SVM and linear regressions using 60 measurements of F_430_/F_580_ at a given X‐ray dose. As illustrated in Figure 6g, both the SVM and linear regression delivere inadequate results in the 0–8 Gy range, where R^2^ < 0.2 and $\bar{R^{2}}$ < 0. This outcome may be attributed to a weak sensitivity of disulfide bonds to X‐rays. Our results clearly indicate that the NPR‐SS/PVA hydrogels can effectively detect X‐rays in the 10–80 Gy range. As shown in Figure 6h,i, the performance metrics (R^2^, $\bar{R^{2}}$, RMSE, and RMSE’) are favorable. Moreover, the SVM regression delivered a higher R^2^ and $\bar{R^{2}}$, lower RMSE and RMSE’, indicating that this approach serves as a more reliable model than linear regression.

**Figure 7:**
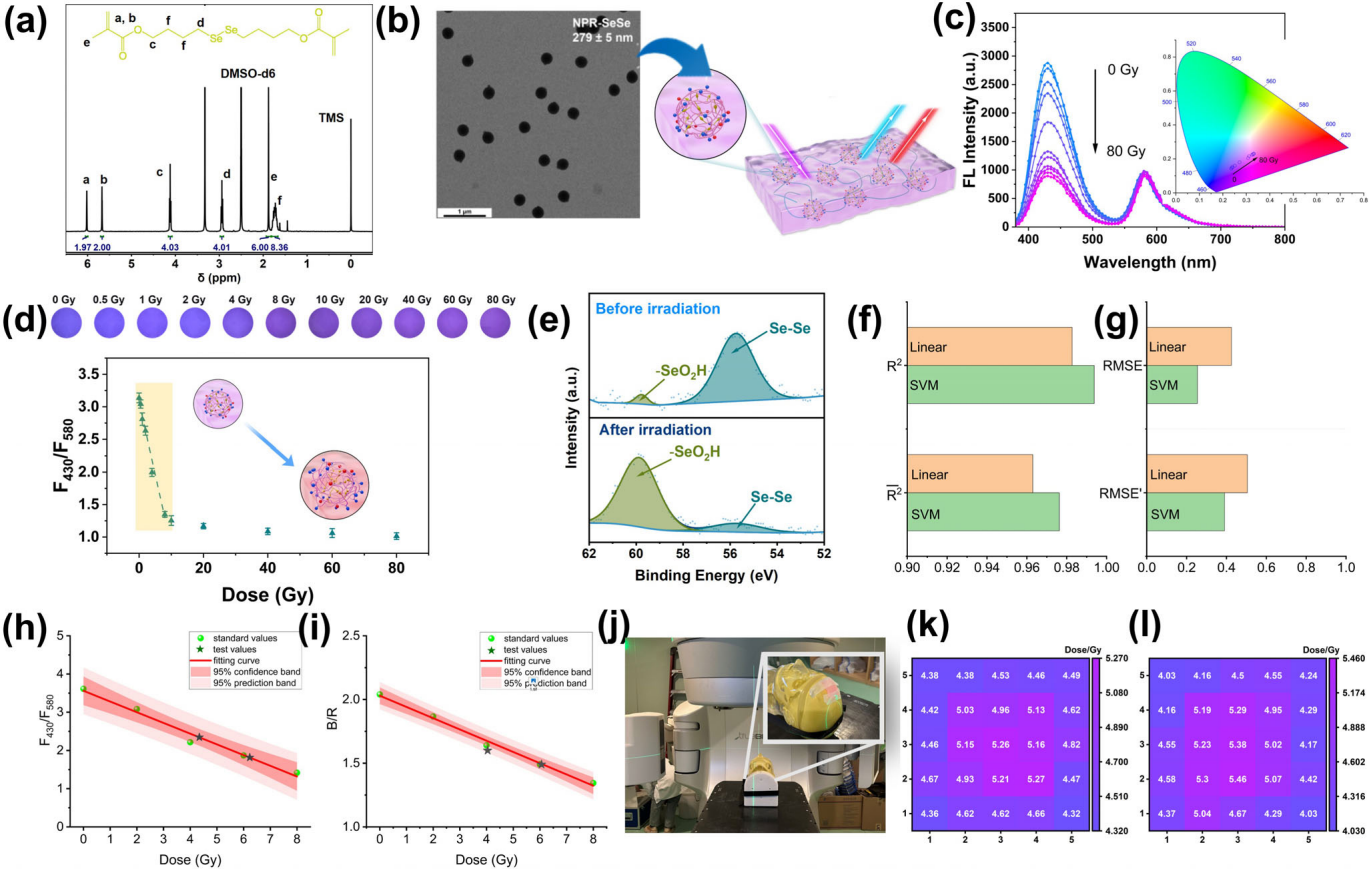
Properties of the NPR‐SeSe/PVA hydrogel responding to X‐rays. a) ^1^H NMR of BMASe, b) TEM image of the NPR‐SeSe microgels and schematic illustration of structure of the NPR‐SeSe/PVA hydrogel, c) Change in FL intensity and CIE image, d) F_430_/F_580_ and fluorescence images of the hydrogel as a function of increasing X‐ray doses. Each data point in panel *d* is the average of 60 measurements, with the error bars showing the standard deviation. e) XPS spectra of the NPR‐SeSe microgels before and after irradiation. f) R^2^ and $\bar{R^{2}}$ and g) RMSE and RMSE’ analysis of the NPR‐SeSe/PVA hydrogel under 0–8 Gy. h) Change in F_430_/F_580_ and i) B/R of the hydrogel as a function of increasing clinical X‐ray doses. Using the hydrogel to map the dose distribution at the forehead skin during a single‐fraction 6 Gy radiotherapy session. j) The experimental setup, dose distribution obtained by fluorescence k) spectra, and l) image analysis.

In order to measure the X‐ray doses in a single‐fraction radiotherapy treatment, it is necessary to improve sensitivity and lower the LOD. For this purpose, we have synthesized a novel diselenium‐containing crosslinker. In this synthesis, a combination of selenium powder, 4‐bromo‐1‐butanol, and NaBH_4_ was heated to give 4‐[(4‐hydroxybutyl)diselenyl]butan‐1‐ol (2SeOH). The resultant 2SeOH was reacted with methacryloyl chloride under N_2_ gas in an ice bath, yielding diselenediylbis (butane‐4,1‐diyl) bis(2‐methylacrylate) (BMASe, Scheme 1; Figure S17a, Supporting Information). The ^1^H NMR and liquid chromatography‐mass spectrometry (LCMS) data confirmed that BMASe was successfully synthesized (**Figure** **7**a; Figure S17b, Supporting Information). Following this, a poly(NIPAm‐co‐PhAN‐co‐RhB‐APMA) (denoted as NPR‐SeSe) microgel was prepared by precipitation polymerization using BMASe as the crosslinker. The chemical structure of NPR‐SeSe microgel was determined by FTIR and UV–vis analysis (Figure S18, Supporting Information). TEM and DLS measurements (Figure 7b; Figure S19, Supporting Information) have established that NPR‐SeSe microgels exhibit a spherical structure, with D_TEM_ and D_H_ of ≈280 and ≈440 nm (PDI = 0.027), respectively. Moreover, fluorescent spectral analysis revealed a dual emission wavelength for NPR‐SeSe microgels at ≈430 and ≈580 nm (λ_ex_ = ≈370 nm, Figure S20, Supporting Information)

Next, the NPR‐SeSe microgels were embedded in PVA, generating NPR‐SeSe/PVA hydrogels. The resultant hydrogel was utilized to sense X‐ray doses by monitoring F_430_ and F_580_ after 2 h post‐irradiation. Under X‐ray exposure (225 kVp, 26.5 Gy min^−1^), F_430_ of NPR‐SeSe microgels exhibited a sharp decrease, whereas F_580_ was stable (Figure 7c). Likewise, the color red‐shifts as observed in the CIE 1931 plot. When compared with NPR‐SS/PVA, the NPR‐SeSe /PVA hydrogel effectively responds to X‐rays in a lower range (0.5–8 Gy) with an enhanced sensitivity (0.2 Gy^−1^, Figure 7d) where:

(5) $$F_{430}/F_{580}=-0.2\times x_{dose}+3.1\;\left(\;R^{2}=0.978\right)$$

The calculated LOD is 0.51 Gy. An increase in the X‐ray dose from 10 to 80 Gy resulted in a slight decrease in F_430_/F_580_ with an associated sensitivity of 2.9x10^−3^ Gy^−1^. Therefore, it can be seen that the fluorescence image of the NPR‐SeSe/PVA hydrogel changes from blue to purple. The NPR‐SeSe /PVA hydrogel exhibits post‐irradiation stability (for 3 days, Figure S21, Supporting Information), and dose‐rate independence (maximum DRIF = 93%±6.67%, Figure S22, Supporting Information). The predicted X‐ray responsivity of NPR‐SeSe/PVA hydrogel results from a change in the chemical structure of the NPR‐SeSe microgels. As shown in Figure 7e, the Se_3d5/2_ XPS peak of the NPR‐SeSe microgels was recorded at 55.5 eV before irradiation, with a minor ─SeO_2_H component generating a signal at 59.4 eV. After 8 Gy X‐ray irradiation, an estimated 82.45% of the diselenium bonds were oxidized to ─SeO_2_H. In addition, the Raman peak at 292 cm^−1^ due to diselenium bonds was significantly reduced following X‐ray irradiation (Figure S23, Supporting Information). Scission of the diselenium bonds increased D_H_ of the NPR‐SeSe microgels to ≈680 nm (Figure S24, Supporting Information) with an accompanying lower F_430_/F_580_.

The SVM analysis and linear regression were conducted at a given X‐ray dose, delivering a good performance in the 0–8 Gy range in the case of NPR‐SeSe/PVA hydrogels (Figure 7f,g). The SVM regression exhibits better outcomes than linear regression, with higher R^2^ and lower RMSE in both single and 10‐fold cross‐validation experiments. The results demonstrate that SVM serves as a better model for NPR‐SeSe/PVA hydrogels in predicting single‐fraction X‐ray radiotherapy dose levels. However, when the X‐ray doses exceeded 10 Gy, both the SVM and linear regression were unsatisfactory (Table S3, Supporting Information).

In verifying clinical efficacy, NPR‐SeSe /PVA hydrogel (5 × 5 × 0.3 cm) was first exposed to different X‐rays (6 MV, 6 Gy min^−1^, TrueBeam, Varian) in the vertical direction. The hydrogels also exhibit X‐ray responsivity, indicating a radiation energy and dose rate independence. Quantitatively, both R/B and F_430_/F_580_ values decrease linearly with increasing X‐ray dosage from 0 to 8 Gy (Figure 7h,i), where:

(6) $$F_{430}/F_{580}=-0.28\times x_{dose}+3.56\;\left(\;R^{2}=0.978\right)$$

(7) $$B/R=-0.09\times x_{dose}+2.02\;\left(\;R^{2}=0.993\right)$$

The method can be utilized to measure X‐ray dosages by substituting either the F_430_/F_580_ or the B/R values into the fitting Equations 6 and 7. Both methodologies can yield stratified data. For example, when exposed to a 6 Gy X‐ray dosage, the fluorescence and image analysis produced results of 6.23 and 6.06 Gy, respectively. Next, we utilized the hydrogel to map the dose distribution at the forehead skin during a single‐fraction radiotherapy session. The NPR‐SeSe/PVA hydrogel was attached to a LUCY head phantom, as illustrated in Figure 7j. Due to the curvature of the head phantom, TPS was deemed invalid. After administering a dose of 6 Gy, we analyzed the images and fluorescent spectra of the NPR‐SeSe/PVA hydrogel at various locations (5 × 5). By applying the R/B and F/F values from the standard curves, 2D X‐ray dose distributions can be obtained. Both analytical methods produced similar results, thus serving as an effective tool for skin dosing measurements in radiotherapy.

## 3 Conclusion

The AIE monomer, PhAN, has been successfully synthesized. Subsequently, dual‐stimuli‐responsive AIE microgels (or NPR‐based microgels) were prepared incorporating NIPAm, PhAN (λ_ex_/λ_em_: 365/430 nm), and Rhodamine B derivatives (λ_ex_/λ_em_: 365/580 nm) using different crosslinkers. Finally, the NPR‐based microgels were introduced into PVA to produce temperature and X‐ray dual‐responsive NPR/PVA ratiometric fluorescence hydrogels. The introduction of microgels increased hydrogen bonding and π‐OH interactions, and the NPR‐SS/PVA hydrogel exhibited enhanced fracture stress (318.1±13.1 kPa), fracture strain (257.7%±11.5%), and adhesion to a variety of substrates. An increase in temperature (20–45 °C) served to reduce the volume of the NPR‐SS microgels, resulting in an increased fluorescence intensity ratio (F_430_/F_580_) of the NPR‐SS/PVA hydrogel, spectrally and visually. Our system could respond to temperature immediately and exhibited exceptional temperature sensitivity, reaching 0.1 °C^−1^. Furthermore, the NPR‐SS/PVA hydrogel could respond to 5–80 Gy X‐rays with a sensitivity of 0.02 Gy^−1^, which is due to a scission of the disulfide‐containing crosslinkers. In order to measure single‐fraction X‐ray radiotherapy dose level, the NPR‐SeSe/PVA hydrogel was prepared using a novel BMASe as the NPR‐SeSe microgel crosslinker. The NPR‐SeSe/PVA hydrogel dose response range decreased to 0.5–8 Gy with a sensitivity of 0.2 Gy^−1^ due to the lower bond energy of diselenide relative to disulfide bonds. These metrics surpassed previously reported values, including sensing range, sensitivity, and LOD. To enhance spectral accuracy and enable analysis of large‐scale clinical datasets in the future, different regression analyses were considered, where the SVM regression delivered the best performance in the case of temperature and X‐ray dosing on the basis of root mean square error, coefficient of determination, and k‐fold cross‐validation experiments. The proposed NPR/PVA hydrogels exhibit significant potential in accurately sensing skin temperature and mapping radiotherapy dose levels. In the future, we will integrate the fluorescent hydrogel into a wearable device, while increasing data volume and optimizing models to improve sensing performance.

## Conflict of Interest

The authors declare no conflict of interest.

## Supporting information



Supporting Information

## Data Availability

The data that support the findings of this study are available from the corresponding author upon reasonable request.
