# Supplementary material for: Multifunctional Fluorescent Microgel‐Embedded Hydrogels for Temperature and X‐Ray Sensing
Source: Adv Sci (Weinh). 2025 Sep 30;12(47):e12518. doi: 10.1002/advs.202512518 (PMC12713054; doi:10.1002/advs.202512518)
Supplement: Supplementary file 1 — Supporting Information [file ADVS-12-e12518-s001.docx]

**Multifunctional Fluorescent Microgel-Embedded Hydrogels for Temperature and X-ray Sensing**

Hao Jiang,^1, ⋕^ Luyao Wang,^2, ⋕^ Li Jiang,^3, ⋕^ Yajie Wang,^2^ Jinlong Zhang,^2^ Mingyuan Pan,^3^ Rui Hu,^4, *^  Jun Li,^5, *^ Chouwang Li,^5^ Chan Kyung Kim,^6^ Hong Chen,^1, *^ Liang Hu^2, *^

1. Key Laboratory of Polymeric Material Design and Synthesis for Biomedical Function, College of Chemistry, Chemical Engineering and Materials Science, Soochow University, Suzhou, China

2, State Key Laboratory of Radiation Medicine and Protection, Collaborative Innovation Center of Radiological Medicine of Jiangsu Higher Education Institutions, and School for Radiological and Interdisciplinary Sciences (RAD-X), Soochow University, Suzhou, China

3. Radiation Oncology Center, Huashan Hospital, Fudan University, Shanghai, China

4. Department of Radiation Oncology, The Affiliated Suzhou Hospital of Nanjing Medical University, Suzhou Municipal Hospital, Suzhou, China

5. School of Chemistry and Chemical Engineering, North University of China, Taiyuan, China.

6. Department of Chemistry and Chemical Engineering, Inha University, Incheon 22212, Korea.

*: Corresponding authors

⋕: These authors contributed equally to this work.

**1. Experimental Section**

***Chemical and Reagents:***

N-Isopropylacrylamide (NIPAm, ≥ 98.0%), N, N'-bis(acryloyl)cystamine (BAC, ≥ 98.0%), 4-bromo-1-butanol (≥ 80.0%)， 1-ethyl-3-(3-dimethylaminopropyl)carbodiimide hydrochloride (EDC, ≥ 98.0%) and cetyltrimethylammonium bromide (CTAB, ≥ 98.0%) were purchased from Tokyo Chemical Industry Co., Ltd (Tokyo, Japan). N, N'-methylenebisacrylamide (BIS, ≥ 98.0%) and deuterochloroform (CDCl_3_, ≥ 99.8%) were bought from Sigma-Aldrich Shanghai Trading Co., Ltd (Shanghai, China). 4-bromo-1,8-naphthalic anhydride (≥ 95.0%), allylamine hydrochloride (≥ 98.0%), phenol (≥ 99.0%), sodium hydroxide (NaOH, ≥ 99.5%), triethylamine (TEA, ≥ 99.0%), methacryloyl chloride (≥ 95.0%), rhodamine B (RhB, ≥ 98.0%), N-hydroxybutanediimide (NHS, ≥ 99.5%), N-(3-aminopropyl)methacrylic acid hydrochloride (APMA, ≥ 98.0%), polyvinyl alcohol 1799 (PVA), ethanol (EtOH, 75%), deuterated dimethyl sulfoxide (DMSO-d6, ≥ 99.9%), and deuterium oxide (D_2_O, ≥99 atom% D) were provided by Shanghai Aladdin Chemical Reagent Co. (Shanghai, China). Anhydrous potassium carbonate (K_2_CO_3_, ≥ 98.0%), anhydrous sodium sulfate (Na_2_SO_4_, ≥ 99.0%), sodium chloride (NaCl, ≥ 99.8%), and methyl tert-butyl ether (MTBE, ≥ 99.0%) were obtained from Shanghai Titan Technology Co (Shanghai, China). Anhydrous magnesium sulfate (MgSO_4_, ≥ 98.0%), anhydrous EtOH (≥ 99.7%), dimethyl sulfoxide (DMSO, ≥ 99.5%), anhydrous methanol (MeOH, ≥ 99.5%), dichloromethane (≥ 99.5%), ethyl acetate (≥ 99.0%) and petroleum ether (PE, ≥ 99.9%) were purchased from Jiangsu Qiangsheng Functional Chemical Co. (Jiangsu, China). Tetrahydrofuran (THF, ≥ 99.5%), sodium borohydride (NaBH_4_, ≥ 97.0%), and anhydrous ether (≥ 99.7%) were acquired from Jiangsu Yonghua Chemical Technology Co. (Jiangsu, China). Selenium powder (≥ 99.9%) was bought from Shanghai J&K Scientific Co., Ltd (Shanghai, China). 2,2’-azobis(2-methylpropionamidine) dihydrochloride (V50, ≥ 99.0%) was obtained from Shanghai Yuanye Bio-Technology Co. (Shanghai, China). 4-Morpholineethanesulfonic acid hydrate (MES, ≥ 99.9%) and phosphate buffered saline (PBS, ≥ 99.9%) solutions were bought from Beijing Soleberg Technology Co. (Beijing, China). Deionized (DI) water was filtered to have a resistivity of 18.25 MΩ⋅cm by a Milli-Q Plus system (Millipore Co. US). Whatman #1 paper filters were obtained from GE Healthcare (Maidstone, UK).

***Synthesis of 4-bromo-N-allyl-1,8-naphthalenedicarboximide (ND-Br)***

Allylamine hydrochloride (15.5 mmol), 4-bromo-1,8-naphthalenedicarboxylic anhydride (15 mmol), and anhydrous EtOH (80 mL) were added to a three-neck flask. The mixture was heated to 80 °C under stirring and refluxed for 8 h. Afterwards, it was allowed to cool to room temperature and filtered.

***Synthesis of 4-phenoxy-N-allyl-1,8-naphthalenedicarboximide (PhAN)***

ND-Br (2 mmol), phenol (4 mmol), K_2_CO_3_ (0.6 mmol), and DMSO (40 mL) were added to a three-necked flask. The reaction was heated to 80 °C and stirred under N_2_ for 12 h. After cooling, the reaction was terminated by adding 250 mL of DI water to the reaction solution. The mixture was extracted three times with dichloromethane, and the organic phases were combined and washed with NaOH (1 mg/mL) to remove residual phenol. Then, the mixture was dried with MgSO_4_ and filtered.

***Synthesis of 4-[(4-hydroxybutyl)diselenyl]butan-1-ol (2SeOH)***

NaBH_4_ (109 mmol) and selenium powder (54.5 mmol) were added to a three-necked flask under N_2_ with an ice bath. Then DI water (300 mL) was slowly added while stirring continuously for 10 min. Following this, an additional amount of selenium powder (54.5 mmol) was added. The mixture was then heated up to 90 °C and stirred continuously for 30 min. It was allowed to cool to room temperature, resulting in a sodium diselenide (Na_2_Se_2_) solution. Next, 4-bromobutanol (130 mmol, dissolved in 50 mL DI H_2_O) was introduced, and the reaction mixture was stirred for 3 h. After extraction with anhydrous ether and ethyl acetate, the mixture was purified, yielding 2SeOH.

***Synthesis of diselanediylbis(butane-4,1-diyl) bis(2-methylacrylate) (BMASe)***

2SeOH (11.1 mmol), TEA (26.6 mmol), and THF (50 mL) were added to a three-necked flask under N_2_ and ice bath conditions. Methacryloyl chloride (26.6 mmol) was added dropwise with stirring, and the reaction was quenched by adding DI water (150 mL) after 6 h. The organic phases were collected after extraction with dichloromethane (3 × 100 mL) and were washed with saturated NaCl solution. The organic layer was then dried and purified to obtain BMASe.

***Synthesis of p(NIPAm-co-PhAN-co-RhB-APMA) (NPR) microgels***

Rhodamine B (0.4 mmol) was added to 5 mL of MES buffer solution, followed by adding EDC(1 mmol) and NHS(0.5 mmol) to activate Rhodamine B. The mixture was stirred at room temperature for 30 min. APMA solution (0.4 mmol, dissolved in 20 mL of PBS buffer solution) was then added, and the mixture was stirred at room temperature for 3 hours to obtain the RhB-APMA solution. The RhB-APMA solution was stored at 4 °C prior to use.

Next, 12 mmol of NIPAm, 0.7 mmol of cross-linking agent (BIS, BAC, or BMASe, BAC and BMASe dissolved in 1 mL MeOH), 0.03 mmol of CTAB, 0.03 mmol of PhAN (dissolved in 1 mL MeOH), and 1 mL of the previously prepared RhB-APMA solution were mixed with 96 mL of DI water. After 1 h of continuous stirring under N_2_, the temperature was raised to 70 °C, and V50 (0.2 mmol, dissolved in 1 mL of DI water) was added to initiate polymerization. After 4 h, the p(NIPAm-co-PhAN-co-RhB-APMA) microgels were obtained. The microgel solution was then cooled to room temperature and filtered through Whatman #1 filter paper. The microgels were centrifuged at 20,000 rpm (30 min, × 3). Finally, the microgels were lyophilized and stored in the dark at 4 °C.

***Synthesis of pNIPAm (N-SS) microgels***

12 mmol of NIPAm, 0.7 mmol of BAC (in 1 mL MeOH), 0.03 mmol of CTAB, and 99 mL of DI water were mixed and heated up to 70 °C under continuous stirring for 1 h under N_2_. Polymerization was initiated by adding 1 mL of V50 solution (0.2 M). The reaction was continued for 4 h to obtain pNIPAm microgel (N-SS). After cooling to room temperature, the microgel was filtered through Whatman #1 filter paper, then centrifuged (20,000 rpm, 30 min, ×3), lyophilized, and stored in the dark at 4 °C.

***Preparation of NPR/PVA hydrogels***

The PVA solution (0.15 g/mL) was stirred at 95 °C for 3 h. Next, 5 mL of NPR microgel solution (10 mg/mL) was added and vortexed thoroughly. The resulting mixture was poured into a square mold (5ⅹ5ⅹ0.3 cm), and allowed to freeze at -20 °C for 12 h. After that, the mixture was thawed at room temperature for 3 h. This freeze-thawed process was repeated for three cycles, generating NPR/PVA hydrogel.

***Characterization***

^1^H nuclear magnetic resonance (^1^H NMR) was performed on a Bruker Avance NEO spectrometer (400 MHz, Bruker, US) using CDCl_3_/ DMSO-d6 as the solvent. Fourier transform infrared spectroscopy (FTIR) spectra were obtained on VERTEX 70 (Bruker, US). The temperature-dependent FT-IR spectra were recorded using a Thermo Fisher Scientific Nicolet IS50 spectrometer equipped with a 10 cm demountable gas cell. The NPR-SS microgel solution (in D_2_O) was allowed to equilibrate for 5 minutes at 25 °C and 50 °C before spectral collection. Transmission electron microscopy (TEM) images were obtained using HT-7700 (Hitachi, Japan). Scanning electron microscope (SEM) images were obtained using a SEM SU 8600 (Hitachi, Japan). The 785 nm excited Raman spectroscopy was performed using a Confocal LabRAM Soleil spectrometer (Horiba Jobin Yvon, France). X-ray photoelectron spectroscopy (XPS) of specimens was carried out on an ESCALAB 250Xi spectrometer (Thermo Fisher, US). The UV–Vis absorbance and fluorescence spectra were obtained using a Synergy NEO microplate reader (BioTek, US). Unless otherwise specified, UV–Vis absorbance and fluorescence spectra tests were conducted at 25 °C. Electrospray Ionization Time-of-Flight Mass Spectrometry (ESI-MS) was carried out on Xevo G2-XS Tof (Waters, UK). The relative molecular mass of BMASe was determined by high-performance liquid chromatography-mass spectrometry (LC-MS, Agilent 1260, Agilent, US).

***Mechanical properties of*** ***NPR SS/PVA hydrogels***

The NPR SS/PVA hydrogel were cut into a dumbbell shape (GB/T 1040-1992, length (*l*): 50 mm, width (*w*): 8 mm, thickness (*d*): 2-2.5 mm, gauge length (l_0_): 16 mm, inner width (*w*_i_): 4 mm), and the samples were uniaxially stretched at a speed of 100 mm/min at room temperature in air.

The tensile stress (σ) was calculated using the following equation:

$\sigma=\frac{F}{d\times w_{i}} (S1)$

Where, *F* was the loading force on the hydrogel.

The tensile strain (ε) was calculated using the following equation:

$\varepsilon=\frac{\triangle l}{l_{0}}\times100\% (S2)$

Where, Δ*l* was the change in gauge length (mm) of the hydrogel.

The Young's modulus ($E$) was calculated as the average slope over 10-20% of ε from the σ–ε curve.

The fracture energy (U, kJ∙m^-3^) was calculated using the following equation:

$U=\int\sigma_{f}d\varepsilon_{f} (S3)$

Where σ_f_ and ε_f_ were the fracture stress and fracture strain of the hydrogel, respectively.

The cylindrical hydrogel (radius (*R*): 5 mm, height (*h*_0_): 15 mm) was prepared for compressive measurements. The compression speed was controlled at a strain rate of 10% min^−1^. Compression stress (σ_c_) was determined using the following equation:

$\sigma_{\boldsymbol{c}}=\frac{F_{C}}{\pi\times R^{2}}\times100\% (S4)$

Where *F*_c_ is the compressive loading force.

Compressive strain ε_c_ was calculated using the following equation:

$\varepsilon_{\boldsymbol{c}}=\frac{\triangle h}{h_{0}}\times100\% (S5)$

Where Δ*h* is the change in hydrogel height.

The maximum compression was set at 90% strain relative to h_0_.

***Shear adhesion tests of NPR-SS/PVA hydrogels***

Shear adhesion experiments were performed by sandwiching hydrogels (2.5 × 3 × 0.3 cm) between two substrates, including porcine skin, glass, polyethylene (PE) sheet, and metal foil. The adhesion tests were performed using a tensile test machine (TH-8203, TopHung, Suzhou, China) at a strain rate of 5 mm/min. The adhesive strength was calculated by dividing the maximum load by the initial contact area.

***MD Simulation***

The molecule models of PVA and NPR/PVA were constructed using MS software. MD simulations were conducted using the Forcite module in MS. All parameters for molecules were based on the COMPASS III force field. Smart algorithm was used in each structural optimization. The maximum iteration step in amorphous cell construction is 10000. Then, the frame with the lowest total energy was selected for subsequent calculations. The simulation was performed under the NVT ensemble, and the NHL thermostat regulated temperature control. The total simulation time for each case was 5000 ps, the time step was set to 1 fs, and the calculation accuracy was set to fine. Uniaxial tensile tests were conducted on the PVA and NPR/PVA hydrogels using a Perl script. Stress was applied in the X-axis direction, with 0 stress in the Y and Z directions. The strain was increased by 0.02 each time until it reached 2. The corresponding stress–strain curves were obtained to evaluate the mechanical response of the systems.

***Cytotoxicity test of NPR-SS/PVA hydrogel***

HeLa cells were cultured in DMEM cell media with 10% FBS and 1% penicillin-streptomycin. The hydrogels were washed in EtOH and PBS solution and placed in DMEM medium containing 10% fetal bovine serum (FBS) at 37 °C for 24 h. The obtained extracts were filtered using a 0.22 µm membrane. The control group was treated with a DMEM medium containing 10% FBS without hydrogels.

The cells were seeded into 96-well plates (1.0×10^4^ cells/well) and placed in a humidified incubator at 37 °C and 5% CO_2_. After 24 h incubation, DMEM medium was removed and replaced with different extracts (100 μL). After 24 and 72 h co-incubation, the wells were washed with PBS (x3). 10 μL of CCK-8 reagent was added into each well, and after 2 h, the absorbance at 450 nm was recorded. The cell viability (%) was calculated using the following equation:

$$Cell viability \left( \% \right)=\frac{{Abs}_{sample}-{Abs}_{blank}}{{Abs}_{control}-{Abs}_{blank}}\times100\% (S6)$$

Where, Abs_sample_, Abs_control_ are the absorbance with and without hydrogel extracts, respectively. Abs_blank_ is the absorbance without the CCK8 reagent.

HeLa cells were stained with a live/dead staining reagent for 10 min, and the stained Hela cells were imaged by a fluorescence confocal microscope (Olympus IX71, Carl Zeiss, Germany).

***In-vivo biocompatibility evaluation***

Six mice (6-8 weeks old, 20-25 g) were randomly divided into two groups: a control group (n=3) and a hydrogel-treated group (n=3). Twenty-four hours before the experiment, the dorsal skin of all mice was shaved with an electric clipper. The NPR-SS/PVA hydrogel and saline gauze were applied to the skin for 4 h. At 24, 48, 72 h after removal of the material, the erythema, edema and allergic responses were observed by the naked eye. At 72h after removal of the material, tissue samples were excised for hematoxylin and eosin (H&E) and Masson staining to assess histopathological changes. Serum was collected for enzyme-linked immunosorbent assay ELISA quantification of TNF-α and IL-6. The Ethics Committee of Soochow University granted approval for the animal study protocol.

***Quantitative Real-Time PCR (qRT PCR)***

Total RNA was extracted from skin tissues at 72 h after dressing removal using TRIzol reagent and reverse-transcribed into cDNA. Expression levels of TNF-α and IL-6 were quantified by qRT PCR, with GAPDH as the internal control. Relative expression levels were calculated using the 2^−ΔΔCt^ method. The primer sequences are as follows: IL-1β, CCTGTAGCCCACGTCGTAG and GGGAGTAGACAAGGTACAACCC; IL-6: CTGCAAGAGACTTCCATCCAG and AGTGGTATAGACAGGTCTGTTGG.

***Temperature responsivity of*** ***NPR-SS/PVA hydrogels***

The fluorescence emission spectra of NPR-SS/PVA hydrogels were measured using a microplate reader (λ_ex_ = 365 nm, Thermofisher Varioskan Flash, USA) at temperatures ranging from 20 to 45 °C.

The temperature sensitivity of NPR-SS/PVA hydrogels was calculated using the following equation:

$$Temperature sensitivity =\frac{{(F_{430}{/F}_{580})}_{45}-{(F_{430}{/F}_{580})}_{20}}{45-20} (S7)$$

Where, (F_430_/F_580_)_45_ and (F_430_/F_580_)_20_ are F_430_/F_580_ at 45, and 20 ^o^C, respectively.

The NPR-SS/PVA hydrogel was adhered to the outer wall of a glass beaker or the human hand. Fluorescent photos were recorded by iPhone 14 Pro under 365 nm UV irradiation. At the same time, the infrared thermo-images were recorded by a Fotric 225S infrared thermal camera.

***Temperature responsivity of*** ***NPR-SS microgels***

The fluorescence emission spectra of NPR-SS microgels were measured by the microplate reader at 20-45 °C. The hydrodynamic diameter (D_H_) of the microgels was determined with DLS analyzer (Zetasizer Nano ZS90, Malvern, UK) at the same temperature range.

***X-ray responsivity of NPR microgels***

NPR-BIS and NPR-SS microgels (2 mg/mL) were irradiated using a small animal IGRT research system (225 kVp, 13 mA, RAD 225CX, Germany) within the range of 0-80 Gy at the dose rate of 0.44 Gy/s. NPR-SeSe microgels (2 mg/mL) were irradiated using RS2000 X-ray biological irradiator (160 kVp, 25 mA, Rad Source Technologies, FL, USA) within the range of 0–16 Gy at a dose rate of 0.02 Gy/s. All samples were placed perpendicular to the beam axis, and they were preserved in the dark after irradiation. The fluorescence emission spectra of the samples were measured by the microplate reader (λ_ex_ = 365 nm). The D_H_ of the microgels after irradiation were measured by DLS.

***X-ray dose responsivity of NPR/PVA hydrogel***

The NPR/PVA hydrogels were irradiated using a small animal IGRT research system (225 kVp, 13 mA, RAD 225CX, Germany) by predesigned doses. The LOD of X-ray sensing was calculated as follows:

$LOD=\frac{3\sigma}{k} (S8)$

where σ represents the standard deviation of F_430_/F_580_ at *x*_dose_ = 0, and *k* is the slope.

***X-ray dose rate independency of NPR/PVA hydrogel***

The NPR/PVA hydrogels were irradiated 4 Gy X-rays under different dose rates by adjusting tube current.

The dose rate independent factor (DRIF) is defined as:

$DRIF=\frac{{{(F_{430}}/{F_{580})}}_{n}}{{{(F_{430}}/{F_{580})}}_{1.941}}\times100\% (S9)$

where (F_430_/F_580_)_n_ and (F_430_/F_580_)_1.941_ represent the fluorescence intensity ratio of the NPR-SS/PVA hydrogels following irradiation at dose rates of n and 1.941 Gy/min, respectively.

***Machine learning***

The K-nearest neighbors (KNN) regression model predicts values by calculating the distances between the target point and the five nearest neighbors in the training set; K was set at 5 in this study. The random forest regression model is a nonlinear method based on ensemble learning that improves model stability and accuracy by constructing multiple decision trees. In this study, we set up 100 decision trees for independent predictions, and took the average as the final prediction value. The support vector machine (SVM) regression model fits the data by finding the optimal hyperplane in the feature space: we applied the Gaussian kernel function to effectively capture nonlinear relationships. The radial-scale parameter was set to 1 to effectively capture nonlinear relationships while considering global patterns. The penalty coefficient was set to 1.2841 based on the standard deviation of the dependent variable. The epsilon-insensitive loss function was fixed at 0.1284 (equivalent to 10% of the dependent variable’s standard deviation), trading off between noise resistance and prediction accuracy. In terms of optimization, the sequential minimal optimization (SMO) algorithm was used, and the convergence conditions included a maximum iteration limit of 1×10^6^ and a gap tolerance of 1×10^-3^, ensuring a balanced trade-off between accuracy and computational efficiency.

Before model processing, all feature data are normalized to eliminate data bias. We randomly split the dataset into a training set (70%) and a test set (30%). Model training was conducted on the training set, whereas the test set was used to evaluate the prediction accuracy and fitting performance for each model, which was assessed using the root mean square error (RMSE) and coefficient of determination (R²). The RMSE metric reflects the average magnitude of the difference between the model's predictions and the actual values, with lower values indicating a closer agreement between predicted and actual values. The R² metric measures the proportion of the variance in the dependent variable that the model can predict, with values closer to 1 indicating a better fit.

$RMSE=\sqrt{\frac{1}{n}\sum_{i=1}^{n} {(y_{i}-{y'}_{i})}^{2}}$ (S10)

$R^{2}=1-\frac{\sum_{i=1}^{n} {(y_{i}-{y'}_{i})}^{2}}{\sum_{i=1}^{n} {(y_{i}-\bar{y})}^{2}}$ (S11)

$\bar{y}=\frac{1}{n}\sum_{i=1}^{n} y_{i}$ (S12)

In equations 16-18, n represents the number of test samples, $y_{i}$ denotes the actual value of the i-th test sample, ${y'}_{i}$is the predicted value of the i-th test sample, and $\bar{y}$ is the mean of the actual values in the test set.

In order to comprehensively evaluate the models' generalization ability, we conducted k-fold cross-validation experiments. The dataset was divided into 10 folds using k-fold cross-validation with random partitioning for training and validation. In each iteration, k-1 subsets were used for training, and the remaining subset was used for testing; the process was repeated k times. The RMSE and R² were calculated across the k experiments to assess model performance for unseen data.

$\mathrm{MSE}_{i}=\frac{1}{n_{i}}\sum_{j=1}^{n_{i}} {(y_{j}-{y'}_{j})}^{2}$ (S13)

$\bar{\mathrm{MSE}}=\frac{1}{k}\sum_{i=1}^{k} \mathrm{MSE}_{i}$ (S14)

$\mathrm{RMSE}^{’}=\sqrt{\bar{\mathrm{MSE}}}$ (S15)

$\bar{R^{2}}=\frac{1}{k}\sum_{i=1}^{k} {R_{i}}^{2}$ (S16)

Taking the i-th fold validation set, $n_{i}$ represents the number of samples, $y_{j}$ denotes the actual value of the j-th sample, ${y'}_{j}$ is the predicted value of the j-th sample, $\mathrm{MSE}_{i}$ represents the mean square error for the i-th fold validation set experiment, $\bar{\mathrm{MSE}} is the average$MSE from the k-fold cross-validation, $\mathrm{RMSE}^{’}$is the root mean square error from the k-fold cross-validation，${R_{i}}^{2}$ is the coefficient of determination for the i-th fold validation set experiment, $\bar{R^{2}}$ is the coefficient of determination from the k-fold cross-validation, and k denotes the number of folds (10 in this study).

***Statistical analysis***

All data are presented as the mean ± standard deviation. Statistical analysis was performed by one-way ANOVA with Tukey’s multiple comparisons using GraphPad Prism.

.

**2. Results and discussion**

**Figure S1**

The chemical shifts of ND-Br molecules at 4.80, 5.17 (5.37) and 5.99 ppm correspond to the three protons on CH-C*H*₂-N and C*H*_2_=C*H*-, respectively. Multiple protons of the naphthalene ring appear in the range of 7.84 - 8.66 ppm.


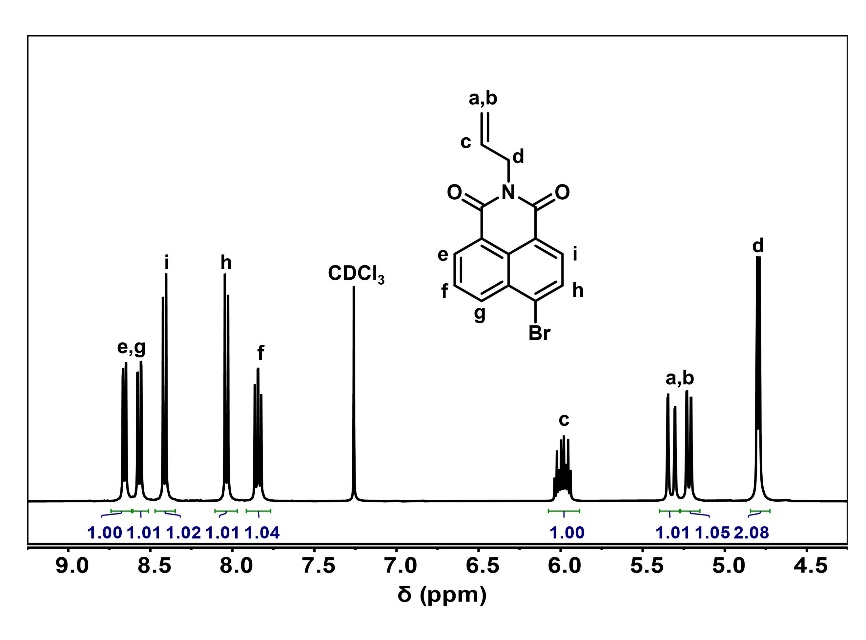


Figure S1. ^1^H NMR spectrum of NB-Br.

**Figure S2.**

**
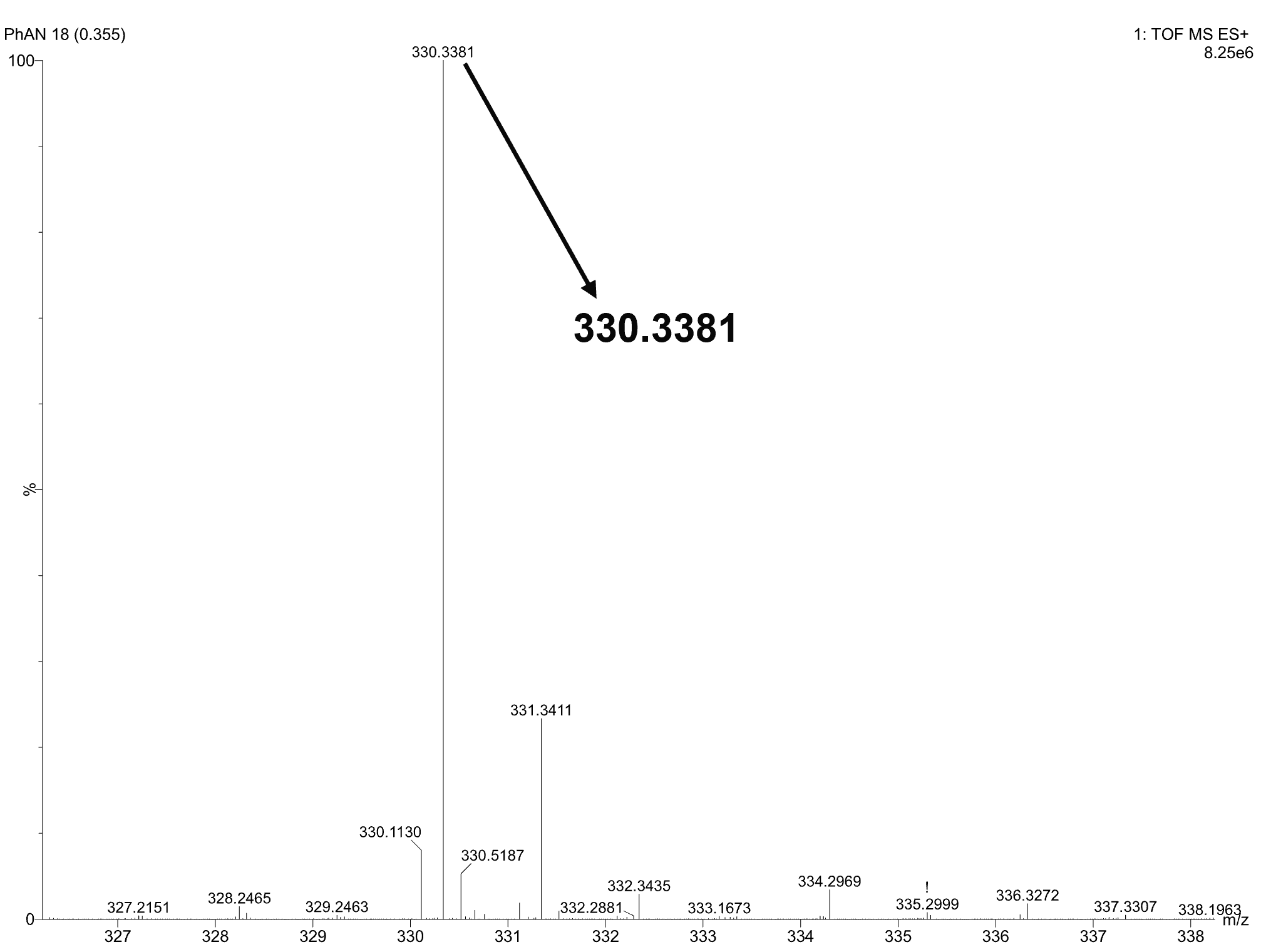
**

Figure S2. ESI-MS spectrum of PhAN.

**Figure S3.**

**
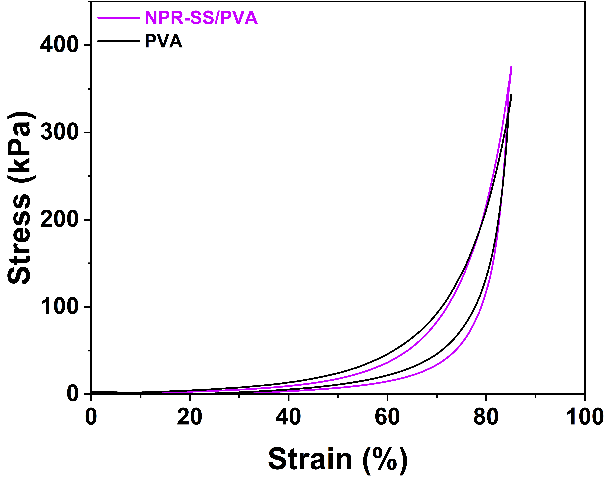
**

Figure S3. Compression property of hydrogels.

**Figure S4**


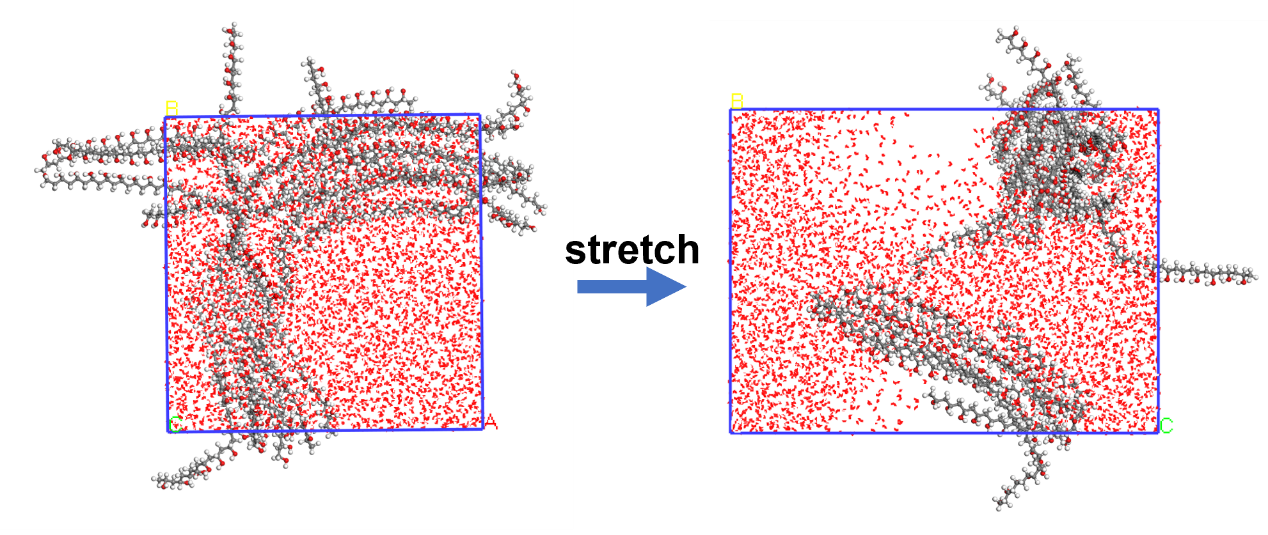


Figure S4. MD simulation and σ-ε curve of the PVA hydrogel.

**Figure S5.**


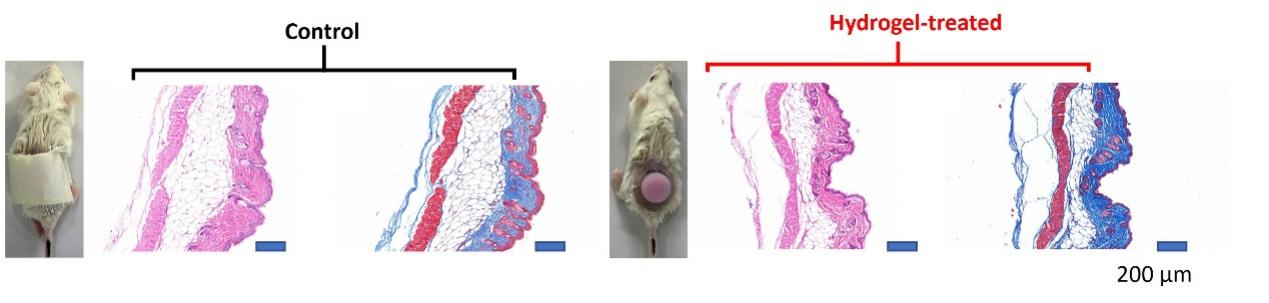


Figure S5. Digital images, Hematoxylin and eosin (H&E), and Masson-stained sections of mice treated with a saline control dressing and the NPR-SS/PVA hydrogel.

**Figure S6**


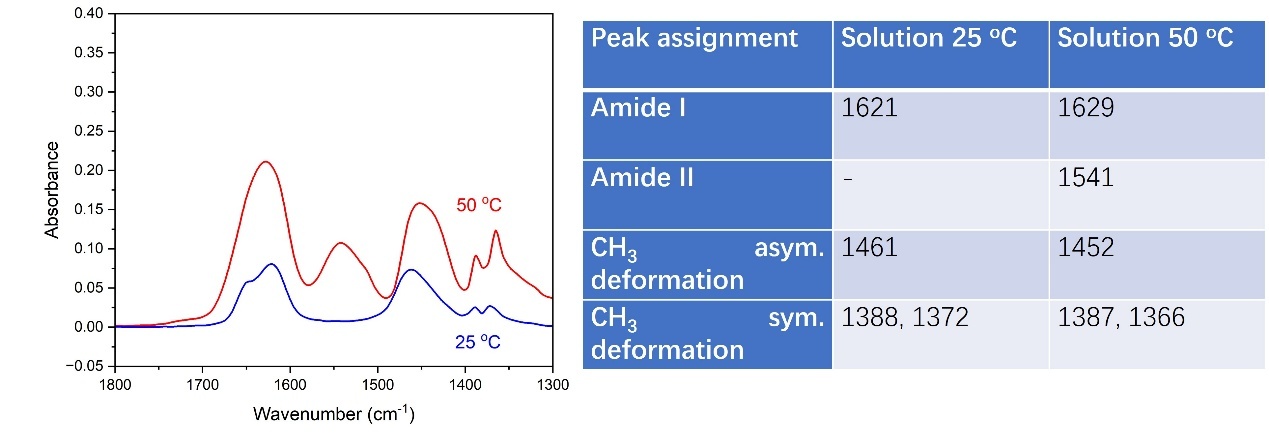


Figure S6. ATR FTIR spectra of the NPR-SS microgel solution in D_2_O at 25 and 50 ^o^C, respectively.

**Figure S7**

An increase in temperature makes NPR-SS microgel deswelling, showing a decrease in D_H_, which can be fitted using the following equation:

$D_{H}=\frac{515.3}{1+exp(\left( x_{temp}-31.3 \right)/dx_{temp})}+375.5$ (S17)

Therefore, fitting the D_H_ at different temperatures using the Boltzmann equation, the LCST_DLS_ of the NPR-SS microgel is 31.3 °C.

**
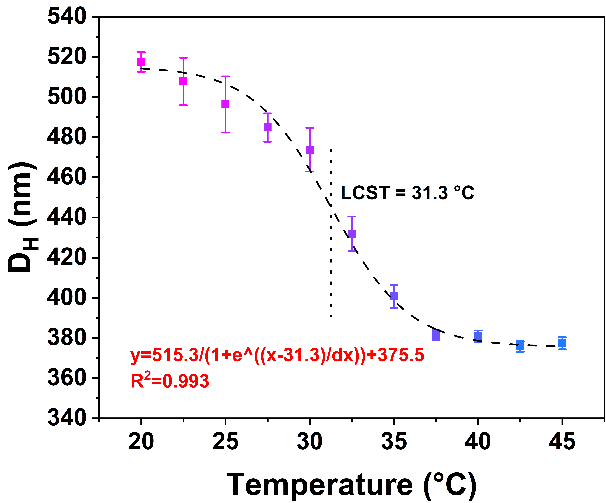
**

Figure S7. Change in D_H_ of NPR-SS microgels as a function of increasing temperature.

**Figure S8**


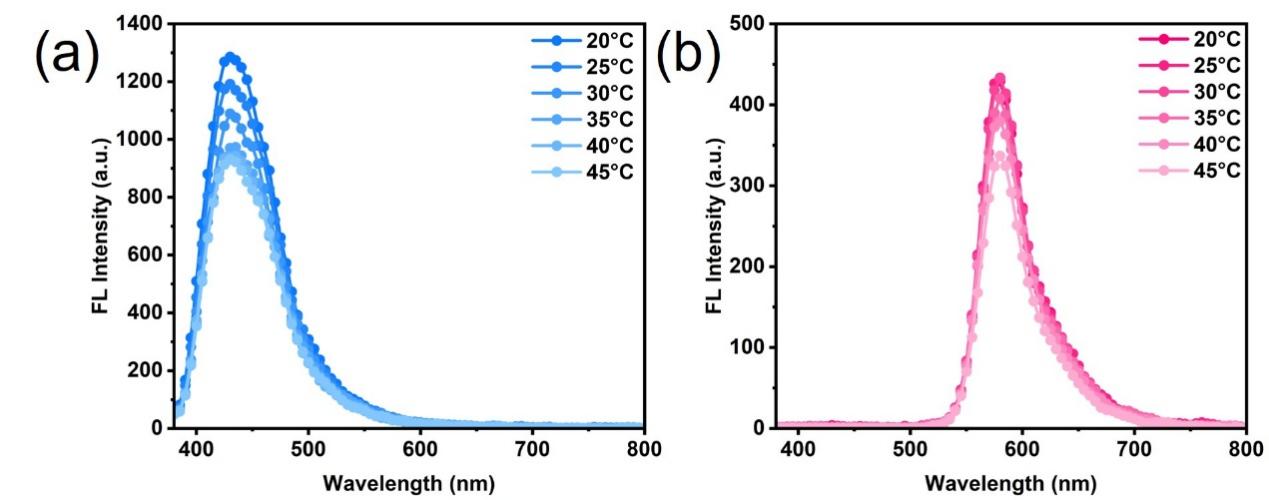


Figure S8. Fluorescence spectra of (a) PhAN in THF (2 mg/mL) and RhB (2 mg/mL) in H_2_O at different temperatures. (λ_ex_: 365 nm).

**Figure S9**

An increase in temperature makes NPR-SS microgel deswelling, showing an increase in F_430_/F_580_, which can be fitted using the following equation:

$F_{430}/F_{580}=\frac{-2.8}{1+exp(\left( x_{temp}-31.5 \right)/dx_{temp})}+5.3$ (S18)

The LCST_FL_ of the NPR-SS microgel is 31.5 °C.

**
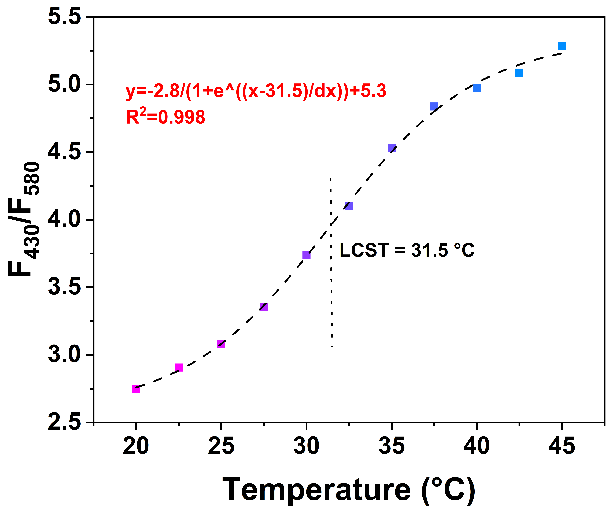
**

Figure S9. Change in F_430_/F_580_ of NPR-SS microgels as a function of increasing temperature.

**Figure S10**

The hydrogel weight (w_g_) was measured at different times. The deswelling ratio (DR) was calculated using the following equation:

$DR=\frac{wg}{{wg}_{0}}\times100\%$ (S19)

where wg_0_ is the initial weight of the hydrogel.


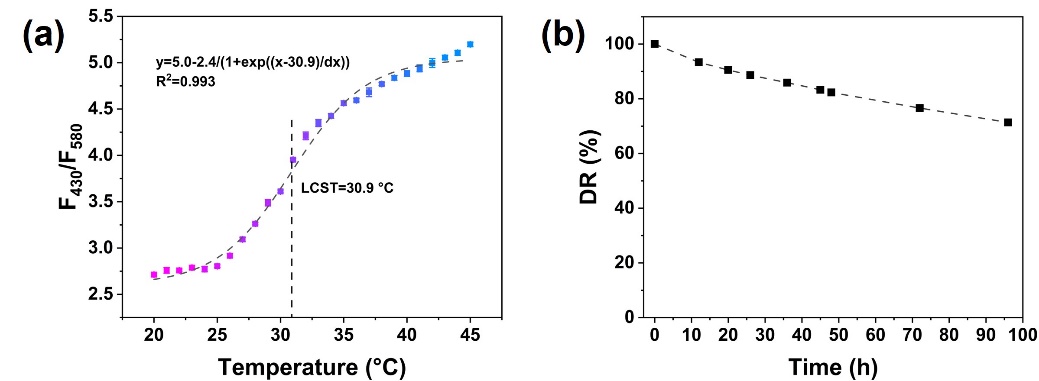


Figure S10. (a) Change in F_430_/F_580_ and (b) DR of NPR-SS/PVA as a function of increasing temperature.

**Figure S11**


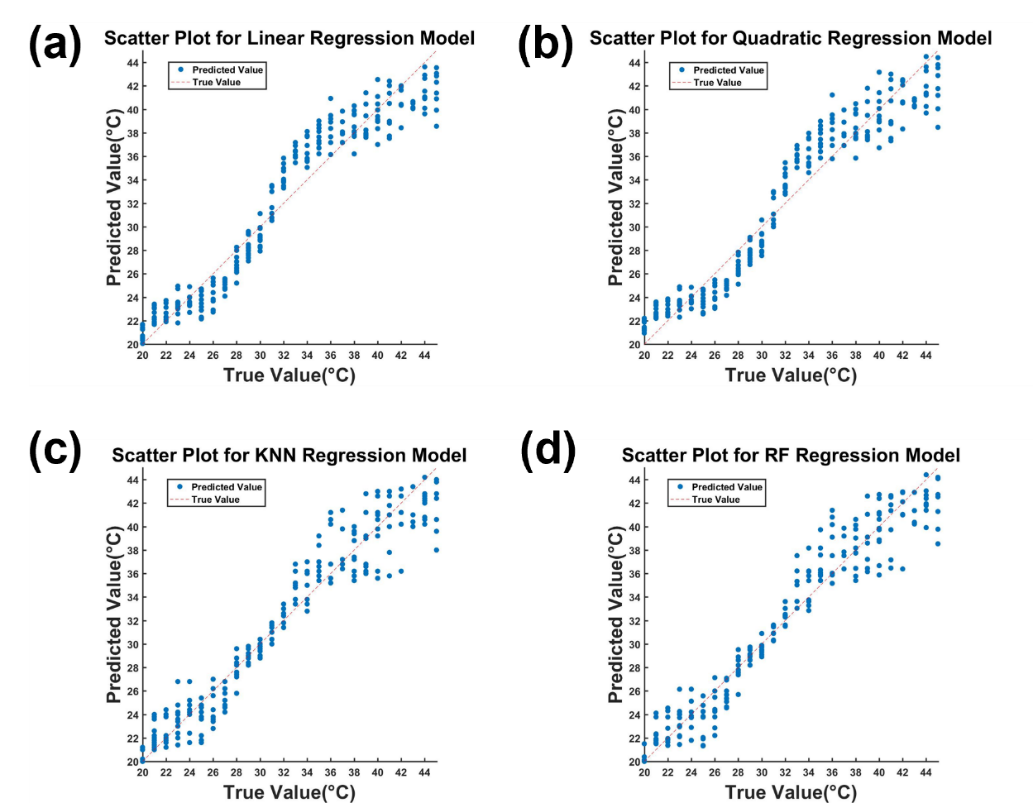


Figure S11. Scatter plots for different regression models.

**Figure S12**


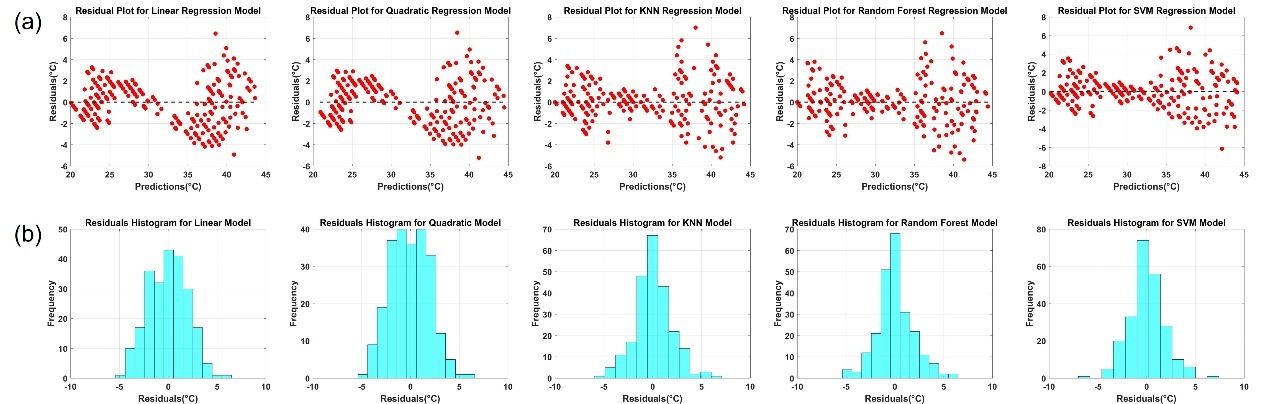


Figure S12. (a) Residual plots and (b) histogram of the residuals for different regression models.

**Figure S13**

**
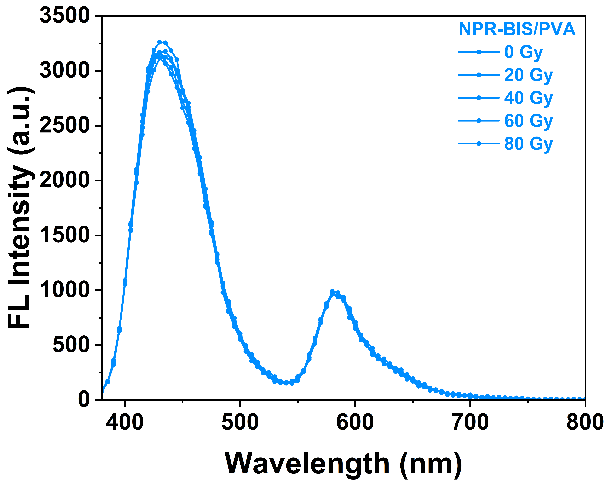
**

Figure S13. FL spectra of NPR-BIS/PVA hydrogel responding to different X-ray doses.

**Figure S14**

**
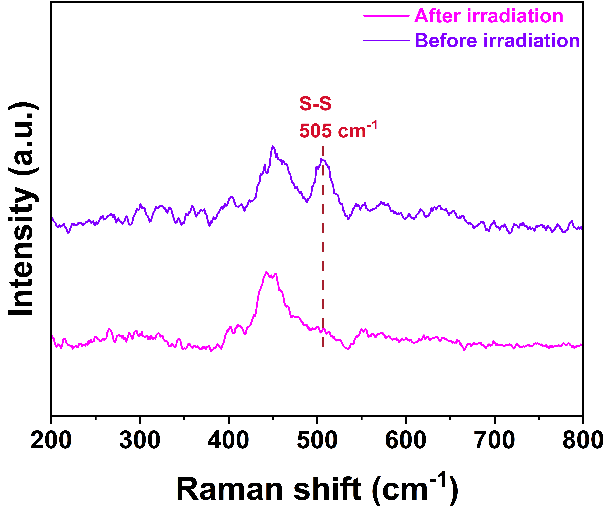
**

Figure S14. Raman spectra of the NPR-SS/PVA hydrogel before and after X-ray irradiation.

**Figure S15**


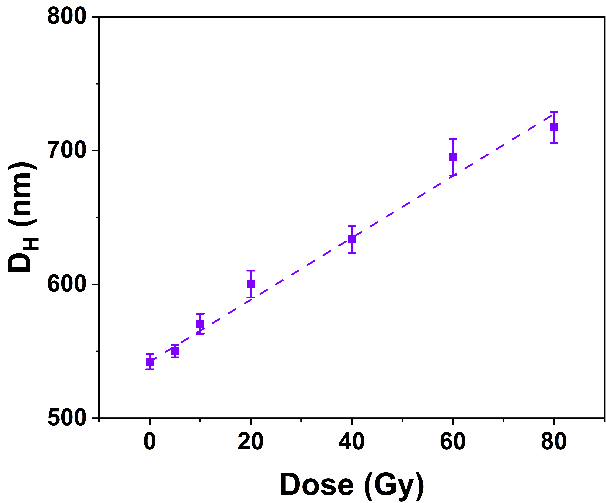


Figure S15. Change in D_H_ of the NPR-SS microgel in responding to different X-ray doses.

**Figure S16**


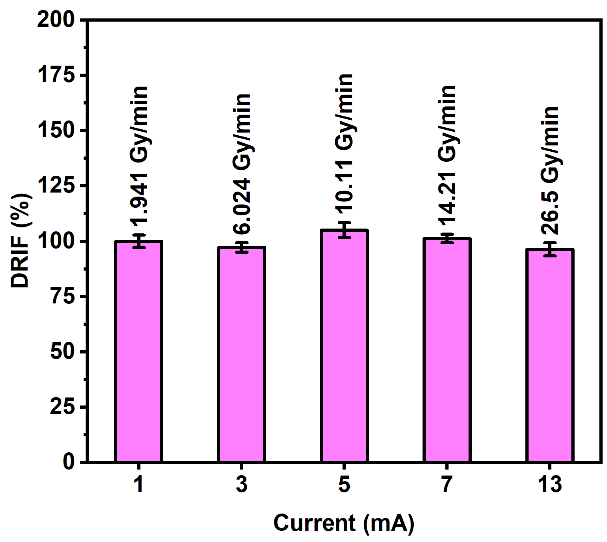


Figure S16. Change in DRIF responding to different X-ray dose-rates.

**Figure S17**.


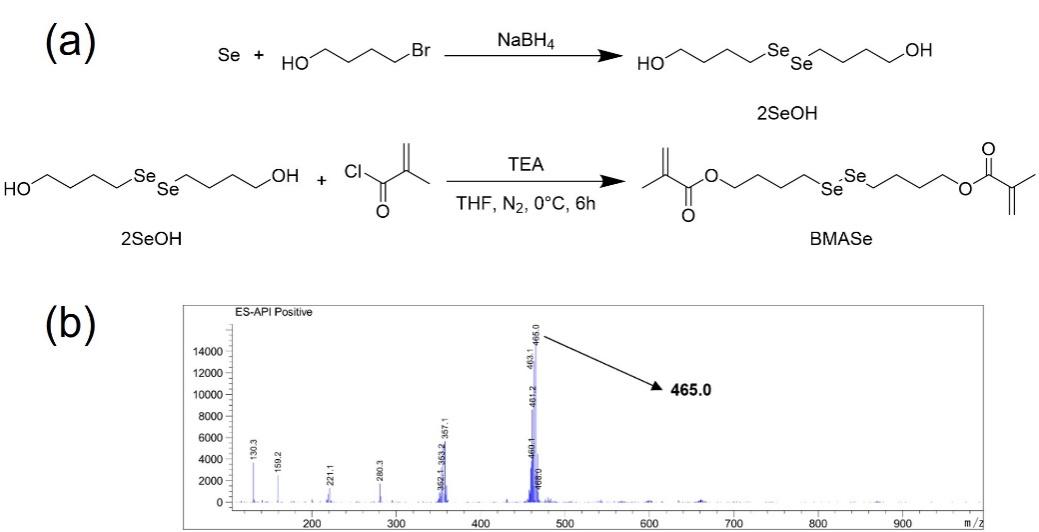


Figure S17. (a) Synthetic routine and (b) LCMS spectrum of BMASe.

**Figure S18**.

The FTIR spectra of the NPR-SeSe microgels exhibit characteristic peaks at 1642 and 1540 cm^-1^ due to amide N-H and C=O stretching vibrations, respectively. The characteristic peak at 1456 cm^-1^ is attributed to a -CH_3_ bending vibration associated with the isopropyl group of NIPAm. The characteristic peak at 1338 cm^-1^ can be ascribed to the C-C stretching vibration of the aromatic backbone in PhAN and RhB-APMA. The NPR-SeSe microgel also exhibits UV-Vis absorption peaks at 365 and 550 nm, respectively, due to the aromatic conjugated structure of RhB and PhAn.


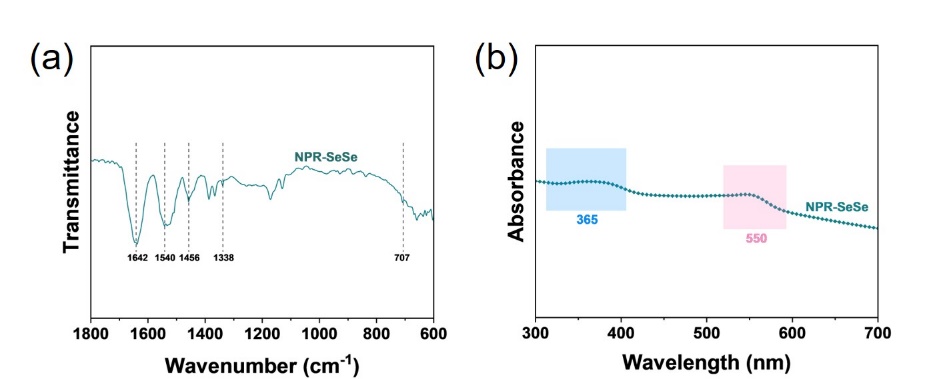


Figure S18. FTIR and UV-Vis spectra of NPR-SeSe microgels.

**Figure S19**.


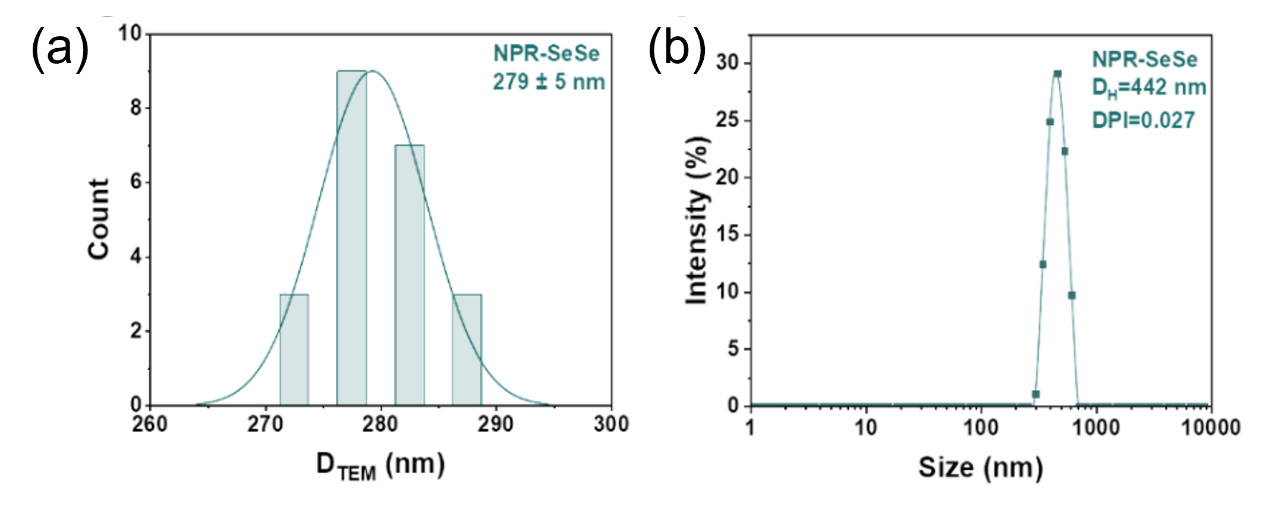


Figure S19. (a) TEM statistical analysis, (b) DLS data of NPR-SeSe microgels.

**Figure S20**.


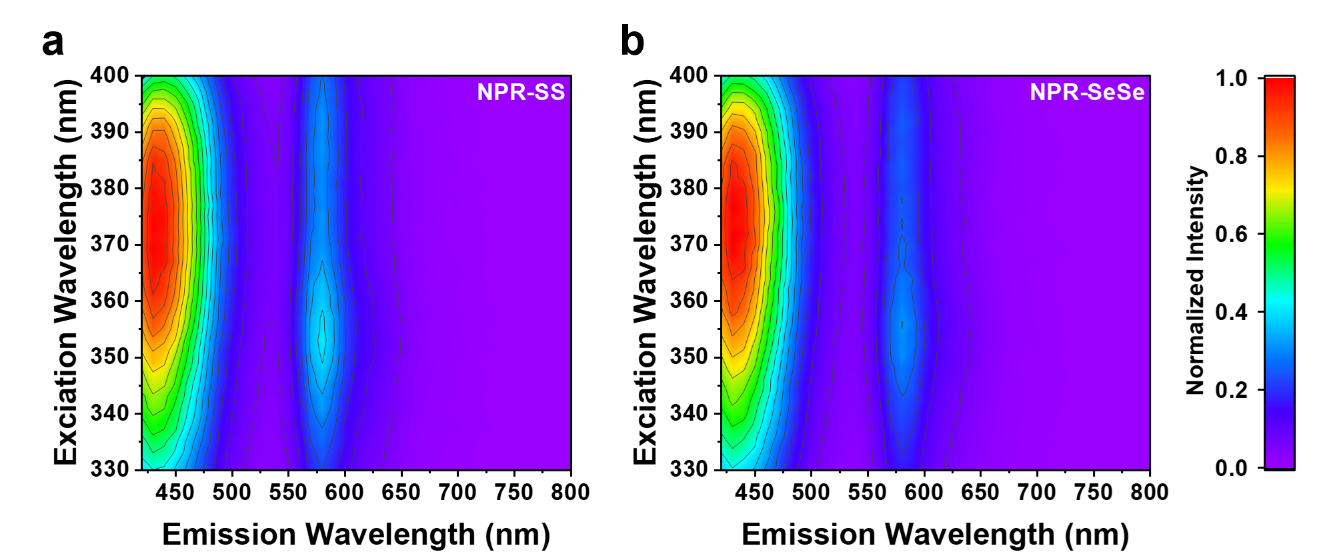


Figure S20. 2D fluorescence emission/excitation spectra.

**Figure S21**.


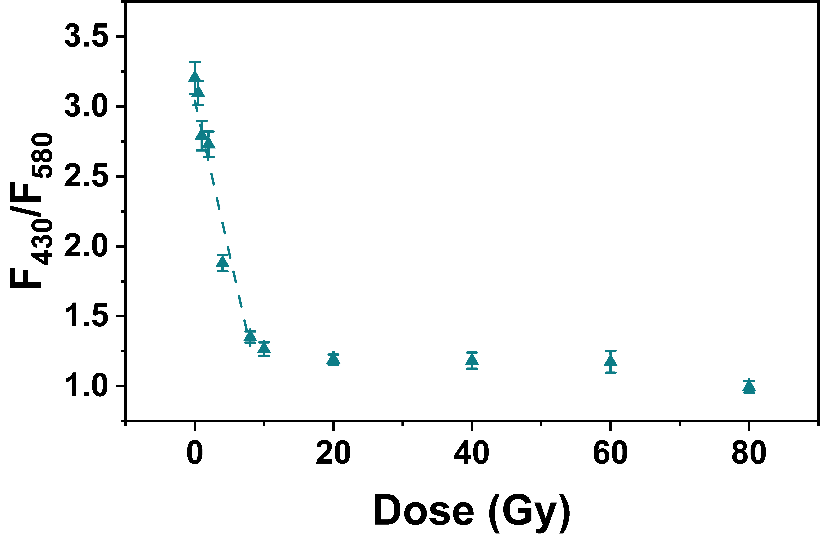


Figure S21. Post-irradiation stability of the NPR-SeSe/PVA hydrogel.

**Figure S22**


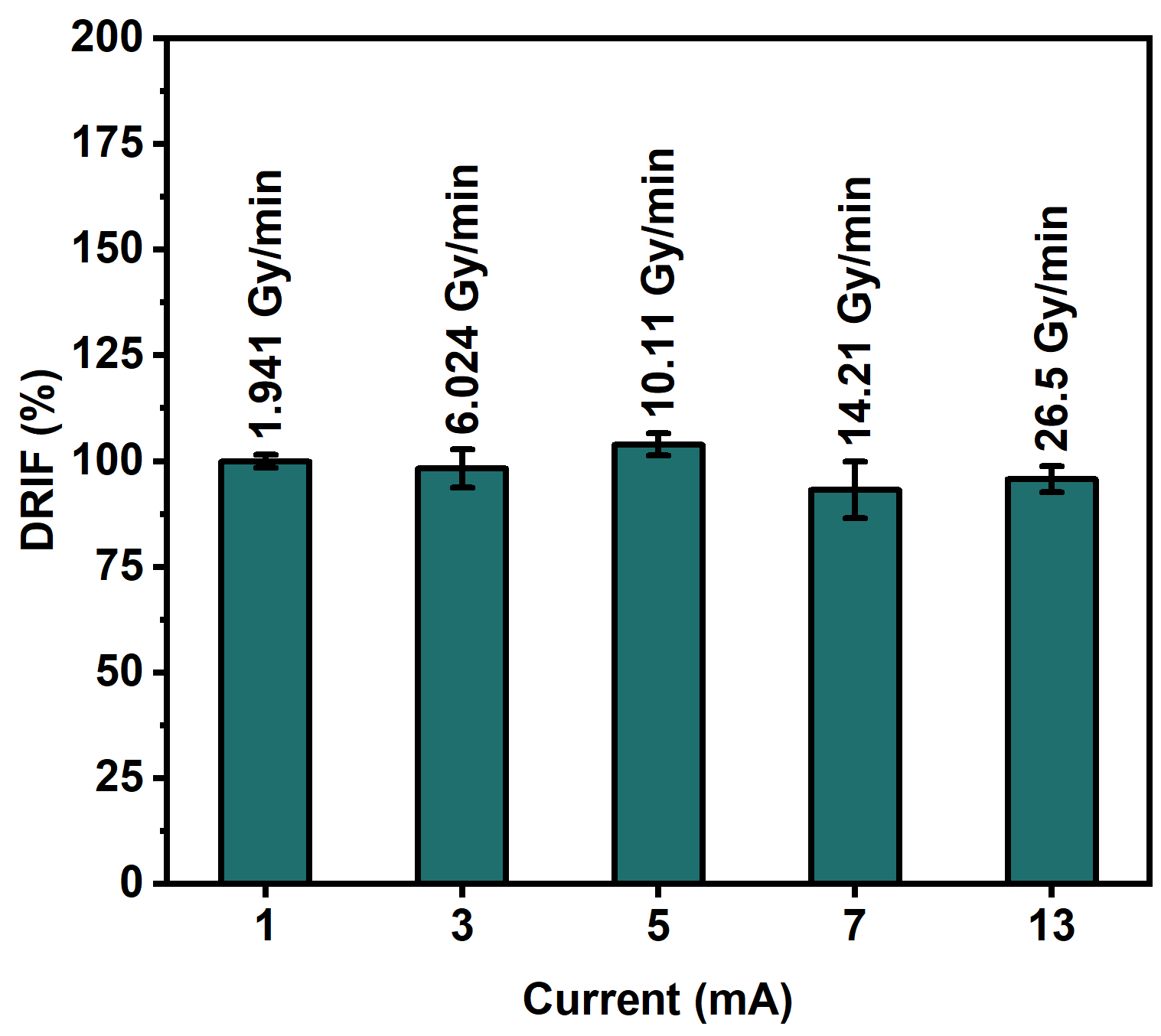


Figure S22. Dose-rate independence of the NPR-SeSe/PVA hydrogel.

**Figure S23**.


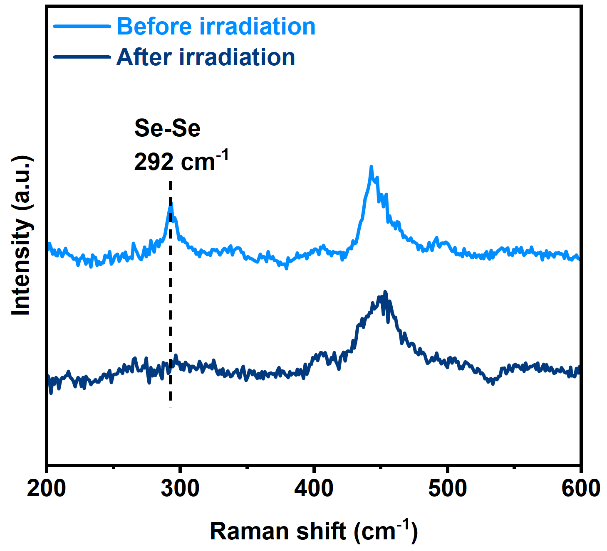


Figure S23. Raman spectra of the NPR-SeSe/PVA hydrogel before and after X-ray irradiation.

**Figure S24.**

**
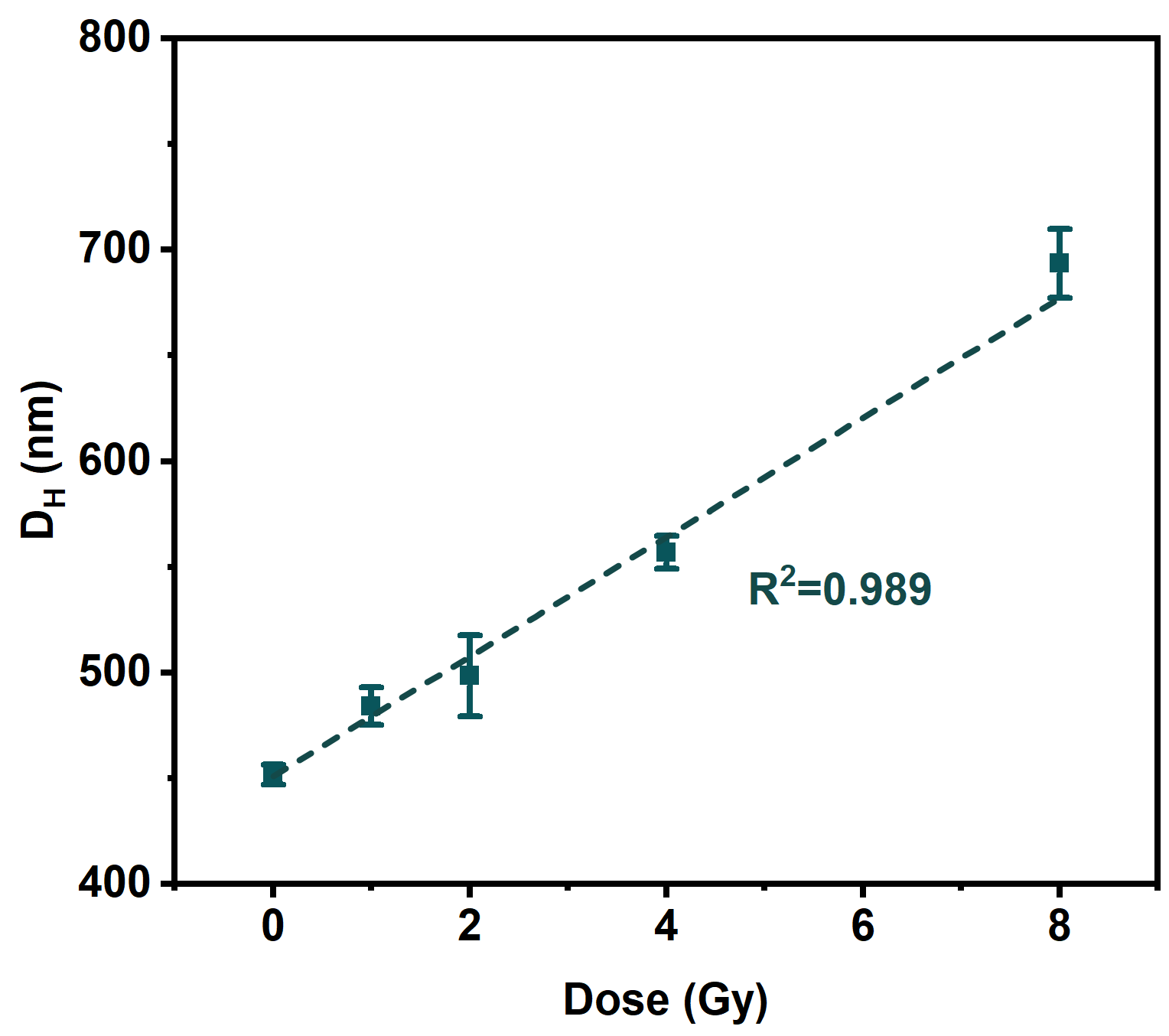
**

Figure S24. Change in D_H_ of the NPR-SeSe microgel in responding to different X-ray doses.

**Table S1**. Temperature-responsive pNIPAm-based fluorescent gels

| Publications | Materials | Fluorescence | Response mechanism | Sensing range/ °C | Sensitivity/°C^-1^ | Mechanical strength  (δ_f_, kPa) | Applications | Other sensing modalities | Machine Learning |
| --- | --- | --- | --- | --- | --- | --- | --- | --- | --- |
| [1] | Nanogel | TPE-based | Dual | 20-50 | 0.005 ^a^ | × | cell imaging | pH | × |
| [2] | Polymeric nanoparticle | TPE-based | Single | 20-50 | 0.2×10^5 b^ | × | cells | × | × |
| [3] | Hydrogel | TPA-based | Single | 20-60 | × | ~70 | Microphase separation | Solvent | × |
| [4] | Microgel | TPE-based | Single | 25-45 | 0.33×10^5 b^ | × | Anti-counterfeiting | Light | × |
| [5] | Nanogel | TPA-based | Single | 23-59 | × | × | Tumor therapy | pH | × |
| [6] | Hydrogel | no | Dual | 25-65 | × | × | Anti-counterfeiting | × | × |
| [7] | Microgel-embedded Hydrogel | no | Dual | 10-50 | 0.06 ^a^ | × | Actuator | pH, Ca^2+^ | × |
| [8] | Polymer solution | TPE-based | Single | 31-38 | 0.44×10^5 b^ | × | cell imaging | Solvent | × |
| [9] | Hydrogel | TPE-based | Dual | 23-70 | 0.019 ^a^ | G’ ≈15 kPa | Anti-counterfeiting | solvent | × |
| ***Our work*** | **Microgel-embedded *Hydrogel*** | ***PhAN***  ***(AIE)*** | ***Dual*** | ***20-45*** | ***0.1*** | **~320** | ***Body temperature*** | ***X-rays*** | ***Yes*** |

TPE: tetraphenylethylene; TPA: triphenylamine; G’: storage modulus;

a: sensitivity is defined as the change in fluorescence intensity ratio divided by the change in sensing temperature;

b: sensitivity is defined as the change in fluorescence intensity at a specific wavelength divided by the change in sensing temperature.

[1] Y. Zhao, et al., ACS Nano 10(6) (2016) 5856-5863.

[2] Z. Wang, et al., Journal of the American Chemical Society 142(1) (2020) 512-519.

[3] Y. Hu, et al., Advanced Materials 33(39) (2021) 2101500.

[4] S. Dong, et al., ACS Applied Materials & Interfaces 14(15) (2022) 17794-17805.

[5] L. Zhang, et al., ACS Nano 17(24) (2023) 25205-25221.

[6] S. Wei, et al., Advanced Materials 35(25) (2023) 2300615.

[7] D. Lu, et al., Advanced Science 11(3) (2024) 2304776.

[8] N. Yin, et al., Journal of Colloid and Interface Science 679 (2025) 519-528.

[9] D. Shi, et al., ACS Nano 19(21) (2025) 19578-19589.

**Table S2. Fluorescent radiation sensors**

| Publications | Materials | Fluorescence | Response Mechanism | Radiation | Sensing range  /Gy | Sensitivity/ Gy^-1^ | LOD  /Gy | Mechanical strength  (δ_f_, kPa) | Biocompatibility | Other sensing modalities | Machine learning |
| --- | --- | --- | --- | --- | --- | --- | --- | --- | --- | --- | --- |
| [1] | Polymer film | Quinoline | dual | γ | 0-4000 | 0.0025 | 80 | × | × | × | × |
| [2] | Molecule solution | TPE (AIE) | single | γ | 0-8 | 50 | 1 | × | × | × | × |
| [3] | Polymer solution | Silole (AIE) | single | γ | 0-40000 | 0.0025 | 130 | × | × | × | × |
| [4] | Molecule solution | Imidazole-based | single | γ | 0-5 | 180 | 0.1 | × | × | × | × |
| [5] | Molecule solution | Imino-BODIPY-based | single | γ | 0-10 | 22.4 | 0.001 | × | × | × | × |
| [6] | Polymer film | Quinoline | dual | X-ray | 0-350 | 0.019 | ~15 | × | × | × | × |
| [7] | Hydrogel | HPF | dual | X-ray/  γ-ray/  e-beam | 0-15 | ~0.2 | 0.5 | × | × | × | × |
| [8] | Nanogel solution | APF | single | X-ray | 0-15 | ~540 | 0.5 | × | × | × | × |
| **This**  **work** | **Microgel-embedded Hydrogel** | **PhAN**  **(AIE)** | **dual** | **X-ray** | **0-80** | **0.02** | **4.9** | **√** | **√** | **√** | **√** |
|  |  |  |  |  | **0-8** | **0.2** | **0.5** |  |  |  |  |

HPF: hydroxyphenyl fluorescein; APF: aminophenyl fluorescein

[1] B. Pei, et al., ACS Applied Materials & Interfaces 12(37) (2020) 42210-42216.

[2] X. Dong, et al., Chemical Communications 51(18) (2015) 3892-3895.

[3] Z. Liu, et al., Journal of Materials Chemistry 21(38) (2011) 14487-14491.

[4] J. M. Han, et al., Journal of the American Chemical Society 136(13) (2014) 5090-5096.

[5] M.K. Choudhary, et al., ChemPhotoChem 8(2) (2024) e202300245.

[6] M. Tang, et al., Nanoscale 17(2) (2025) 846-854.

[7] L. Jiang, et al., Chemical Engineering Journal 459 (2023) 141547.

[8] L. Jiang, et al., ACS Sensors 6(4) (2021) 1643-1648.

**Table S3.** The SVM and linear regression of the NRP-SeSe/PVA hydrogel responding to 10-80 Gy X-rays.

|  | SVM | Linear |
| --- | --- | --- |
| RMSE | 18.42 | 16.73 |
| R^2^ | 0.37 | 0.48 |
| RMSE | 16.87 | 15.87 |
| ‾R^2^ | 0.43 | 0.49 |
